# Balanced mitochondrial and cytosolic translatomes underlie the biogenesis of human respiratory complexes

**DOI:** 10.1186/s13059-022-02732-9

**Published:** 2022-08-09

**Authors:** Iliana Soto, Mary Couvillion, Katja G. Hansen, Erik McShane, J. Conor Moran, Antoni Barrientos, L. Stirling Churchman

**Affiliations:** 1 Blavatnik Institute, Department of Genetics, Harvard Medical School, Boston, MA 02115 USA; 2 Department of Neurology, University of Miami Miller School of Medicine, Miami, FL 33136 USA

## Abstract

**Background:**

Oxidative phosphorylation (OXPHOS) complexes consist of nuclear and mitochondrial DNA-encoded subunits. Their biogenesis requires cross-compartment gene regulation to mitigate the accumulation of disproportionate subunits. To determine how human cells coordinate mitochondrial and nuclear gene expression processes, we tailored ribosome profiling for the unique features of the human mitoribosome.

**Results:**

We resolve features of mitochondrial translation initiation and identify a small ORF in the 3′ UTR of *MT-ND5*. Analysis of ribosome footprints in five cell types reveals that average mitochondrial synthesis levels correspond precisely to cytosolic levels across OXPHOS complexes, and these average rates reflect the relative abundances of the complexes. Balanced mitochondrial and cytosolic synthesis does not rely on rapid feedback between the two translation systems, and imbalance caused by mitochondrial translation deficiency is associated with the induction of proteotoxicity pathways.

**Conclusions:**

Based on our findings, we propose that human OXPHOS complexes are synthesized proportionally to each other, with mitonuclear balance relying on the regulation of OXPHOS subunit translation across cellular compartments, which may represent a proteostasis vulnerability.

**Supplementary Information:**

The online version contains supplementary material available at 10.1186/s13059-022-02732-9.

## Background

Most of the cellular ATP is produced by the oxidative phosphorylation (OXPHOS) system that resides in the inner mitochondrial membrane. The OXPHOS system consists of the four respiratory complexes (Complexes I, II, III, and IV), which create a proton gradient across the membrane that drives the final OXPHOS complex, the ATP synthase (Complex V). These complexes are multimeric, formed by subunits encoded on both nuclear DNA and mitochondrial DNA that assemble together at the inner membrane. While the OXPHOS system is schematically represented as a linear flow involving one copy of each complex, the functional complexes physically associate in subgroups known as supercomplexes that often do not have 1:1 stoichiometry. Indeed, the steady-state levels of the five complexes in bovine heart mitochondria are known to vary greatly, with the levels of the most abundant being six times higher than the least [1, 2].

The mitochondrial DNA (mtDNA) is derived from the α-proteobacterium genome and has slowly reduced in size through 1.5 billion years of evolution within the eukaryotic cell. Of the >1000 proteins that localize to human mitochondria [3], only 13 are encoded on mitochondrial DNA (mtDNA) and synthesized by mitochondrial ribosomes (mitoribosomes) in the mitochondrial matrix. These proteins, which comprise the core subunits of the OXPHOS complexes, are co-assembled with >45 nDNA-encoded OXPHOS subunits that are synthesized by cytosolic ribosomes (cytoribosomes) and imported into mitochondria. Coordinated expression and coregulation of nuclear and mitochondrial genomes maintains mitonuclear balance, in which mtDNA- and nDNA-encoded OXPHOS subunits are in equilibrium, ensuring accurate assembly and proper function of complexes [4–6]. Unassembled subunits can overwhelm proteostasis pathways and assembly intermediates are at risk of producing reactive oxygen species (ROS); both of these scenarios are detrimental to the cell and lead to disease and aging phenotypes [7–11]. In *Saccharomyces cerevisiae*, cytosolic and mitochondrial translation programs occur synchronously during OXPHOS biogenesis [12]. Thus, cross-compartment translation regulation [11] contributes to mitonuclear balance in yeast cells.

Expression and regulation of mtDNA differ substantially between fungal and animal cells. Yeast mt-mRNAs have long 5′ leaders (up to ~1 kb) to which nuclear DNA-encoded translation activators (TAs) bind to recruit mitoribosomes to particular mt-mRNAs, enabling nuclear gene expression to directly control mitochondrial translation [13–16]. However, analogous mechanisms of translational control are not thought to exist in human cells. Human mtDNA molecules are transcribed as two polycistronic transcripts that are processed largely through the excision of mt-tRNAs flanking protein-coding mt-mRNAs, leaving most mt-mRNAs leaderless or with short, 1–2 nt leaders [17, 18]. Most yeast TAs are not conserved in humans, and those that are serve different functions [19]. To date, only one TA for human mitochondrial translation has been identified [20]. Thus, human and yeast cells may use different strategies to balance mitonuclear expression and OXPHOS assembly.

In this study, we re-engineered ribosome profiling to comprehensively capture translating mitoribosomes with subcodon resolution and quantified mitoribosome density across all transcripts in five human cell types. The resultant detailed view of mitochondrial translation across the 13 canonical open reading frames (ORFs) revealed features of translation initiation on leaderless mt-mRNAs. We also discovered a small ribosome-engaged ORF in the 3′ UTR of *MT-ND5* whose translation is retained within the human population. Investigating mitonuclear coregulation across cell types revealed a modest correspondence between the average RNA abundances of nDNA- and mtDNA-encoded OXPHOS subunits. Remarkably, we found that translational control tightened the correspondence substantially, such that average mitochondrial and cytosolic subunit synthesis for each OXPHOS complex demonstrated a near perfect correlation. In contrast to the situation in yeast [12], correlated translatomes in human cells were not achieved through rapid crosstalk between cytosolic and mitochondrial translation systems. Moreover, we found that cytosolic protein synthesis was not affected by a chronic deficiency in mitochondrial protein synthesis induced by the absence of nDNA-encoded protein LRPPRC, which when present stabilizes most mt-RNAs and promotes their polyadenylation and loading onto the mitoribosome [21–25]. Ongoing cytosolic translation despite a repression of mitochondrial protein synthesis resulted in unbalanced translatomes, which lead to the upregulation of proteostasis genes.

## Results

### High-resolution profiling of human mitochondrial translation

**Fig. 1:**
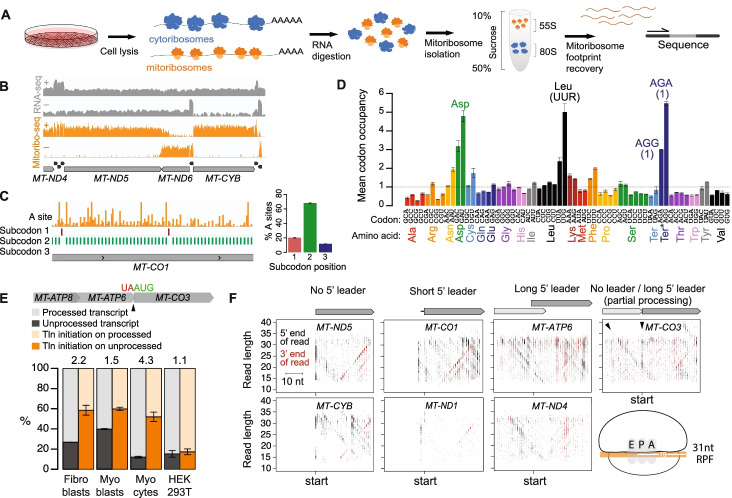
Human mitoribosome profiling reveals features of mitochondrial translation. **A** Schematic of the human mitoribosome profiling approach. **B** Sequencing data over a representative portion of the mitochondrial genome, with mRNAs (gray, labeled) and tRNAs (black) annotated below. Panels show RNA-seq read coverage (gray) and mitoribosome profiling (Mitoribo-seq) read A-site density (orange) on heavy (+) and light (−) strands. *Y* axis is in log scale. **C** Left panel: A-site density (orange) across a portion of *MT-CO1* with subcodon position of maximum signal plotted below. Right panel: Percentage of reads with their A sites on each subcodon position across all mt-mRNAs. **D** Mitoribosome occupancy across codons. Asp, Leu (UUR), and termination codons are labeled. AGA and AGG Ter* putative termination codons are each present only once (indicated in parentheses), at the ends of *MT-CO1* and *MT*-*ND6*, respectively. R: purine (A or G). Codons with occupancy of >3× expected are labeled. Error bars show range across replicates. **E** Percentage of processed and unprocessed *MT-ATP8/6-CO3* transcript at site indicated by arrowhead and of translation (Tln) initiation on processed and unprocessed transcript. Numbers at top of bars indicate the fold-preference for translation initiation on unprocessed vs processed transcripts. Error bars show range across replicates. **F** Scatterplots to visualize mitoribosome position during initiation. Start-codon-aligned RPF 5′ and 3′ ends are plotted according to their genome position and read length. Arrowheads highlight *MT-CO3* initiation on both unprocessed *MT-ATP8/6-CO3* and processed *MT-CO3* mRNA. Cartoon shows positioning of mitoribosome E, P, and A sites on a full-length (31 nt) RPF. Short 5′ leaders consist of one to three nts

The human mitoribosome is highly proteinaceous, containing an RNA:protein ratio of 1:2, opposed to the 2:1 ratio observed in cytosolic human and bacterial ribosomes, and is largely associated with the inner membrane in order to facilitate co-translational insertion of OXPHOS proteins [26]. These unique features make quantitative biochemical purification of mitoribosomes for the purpose of analyzing their translatomes more difficult than the purification of cytosolic ribosomes [27]. To dissect mitochondrial translation with high resolution and minimal bias, we re-engineered ribosome profiling to accommodate the unique characteristics of the human mitoribosome, refining each step of the approach (Fig. 1A).

We first identified lysis and purification conditions that improved the recovery of mitoribosomes by ~2-fold (Additional file 1: Figure S1A). Because RNA absorbance measurements are dominated by cytoribosomes, we used western blot analysis of both the small and large mitoribosome subunits to follow mitoribosome sedimentation (Additional file 1: Figure S1B). We observed that both mitoribosome subunits peaked in a single monosome fraction, followed by a periodic co-sedimentation through later fractions, indicative of polysomes, which have been challenging to capture previously [18, 28]. We did not observe polysomes under non-optimized conditions (data not shown). Thus, our optimized lysis enriches for mitochondrial polysomes (Additional file 1: Figure S1B), permitting a broader view of mitochondrial translation. Finally, RNase treatment revealed a mitoribosome footprint of 31–34 nucleotides (nt) (Additional file 1: Figure S1C,D). We analyzed mitoribosome footprints in five human cell types: primary fibroblasts, primary myoblasts, myocytes (post-differentiation-induction day 2 myoblasts), HeLa S3 cells, and HEK293T cells (Additional file 1: Figure S1E). Sequencing and alignment of mitoribosome footprints revealed specific coverage of mitochondrial coding sequences (Fig. 1B). Our optimized approach consistently yields full-length mitoribosome footprint reads covering >90% of mitochondrial codons for fibroblasts, HeLa S3 cells, and HEK293T cells (Additional file 2: Table S1). This contrasts with previously published protocols for which full-length footprint reads consistently cover <90% of mitochondrial codons. (Additional file 1: Figure S1E, Additional file 2: Table S1). We also achieved exceptional three-nucleotide periodicity in select datasets (Fig. 1C, Additional file 2: Table S1), both signs of high data quality. We also sequenced footprints from mitoribosomes that were purified twice through sequential sedimentation and immunoprecipitation (Additional file 1: Figure S1F), which increased the fraction of reads mapped to mitochondrial mRNA but did not impact data quality (Additional file 2: Table S1).

Due to the high resolution of our optimized approach, we were able to analyze mitoribosome pausing at codon resolution across the cell lines. We detected increased mitoribosome occupancy on several codons, including canonical and noncanonical termination codons (Fig. 1D, Additional file 1: Figure S1G). Pausing at AGA and AGG putative stop codons for *MT-CO1* and *MT-ND6* (Additional file 1: Figure S1H) is consistent with both an induction of frame-shift by these codons to a canonical UAG termination codon [29] and direct termination by an alternative mechanism [30]. Pausing at aspartate (Asp) and leucine (Leu) was pervasive, with mitoribosome stalling observed at nearly every occurrence of either codon (Additional file 1: Figure S1I).

### Resolved features of mitochondrial translation initiation

Most mitochondrial mRNAs are rapidly excised from the long nascent polycistronic transcript that leave leaders of various lengths and many with no leader at all [18]. Translation initiation occurs from the canonical AUG but also AUA and AUU. Initiation on leaderless mRNAs has been proposed to be more efficient than on those with even a short leader [31], but in general, we found that the length of the 5′ leader did not correlate with translation efficiency (Additional file 1: Figure S1J), with one interesting exception. Unprocessed *ATP8/ATP6-CO3* mRNA accumulates to higher levels than other processing intermediates [32] and we observed *MT-CO3* translation initiation on both processed and unprocessed transcripts (Fig. 1E). The frequency of *MT-CO3* mRNA translation initiation varied across cell types but was up to 4.3-fold higher on unprocessed transcripts (Fig. 1E). Thus, for *MT*-*CO3* mRNA, the presence of a leader sequence favors translation initiation.

To analyze mitochondrial translation initiation on mRNAs with minimal or no 5′ UTRs, we visualized mitoribosome positions during initiation by analyzing ribosome-protected fragment (RPF) position versus RPF size (Fig. 1F). *MT-ATP6* and *MT-ND4* mRNAs have long leaders, so the high ribosome density at the start codon is visualized as a V, due to some reads arising from over- or under-digested RPFs. For mRNAs with a concise (1–3 nt) leader or no (0-nt) leader, the plots reveal an enrichment of 15-nt half-size RPFs increasing in size to full length, at which point the mRNA channel is fully occupied. These shorter half-footprints have not been previously detected [33] and indicate that mitoribosomes load at the ends of messages with the P site on the start codon. The clearly discernible pattern of short RPFs increasing to full-length RPFs suggests that the mitoribosome proceeds slowly after initiation until the mRNA channel is fully occupied.

### Mitoribosome-engaged noncanonical open reading frame

**Fig. 2:**
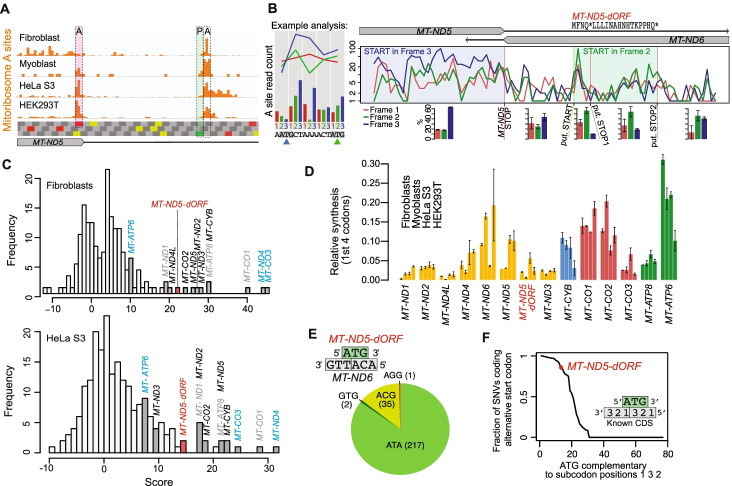
Evidence for the translation of a novel mitochondrial noncanonical open reading frame. **A** Translation initiation occurs on an internal AUG in the *MT-ND5* 3′ UTR across cell lines. A-site density on the *MT-ND5* stop codon (shaded in red) and P-site density (shaded in green, A-site density one codon downstream) of initiating ribosomes on the internal AUG are highlighted. Codons in the three frames are shown below with stop (UAA, UAG), start (AUG), and mitochondrial alternative start (AUU, AUA) highlighted (red, green, yellow, respectively). **B** Stacked frame plot highlighting number of A-site transformed reads in each subcodon position on heavy strand. The example analysis (left panel) shows how raw A site read counts are transformed to line plots, and how the line plots highlight which frame is being read. The color of the arrowheads indicates which frame each ATG is in. The blue ATG starts an open reading frame. Mitoribosome profiling data are from two fibroblast replicates, summed. Amino acid sequence of putative MT-ND5-dORF peptide is shown with putative (put.) start and stop codons highlighted with green and red lines, respectively. Bar plots below show the average percentage of reads in each subcodon position for regions indicated, with error bars showing range between the replicates summed above. **C** Distribution of initiation scores after applying ad hoc scoring method to fibroblasts and HeLa S3 cells. Gene names in black: known genes with no leader; gray: known genes with 1 to 3 nt leader; cyan: known genes with long 5′ UTR. **D** Estimated relative synthesis of *MT-ND5-dORF* compared to other mtDNA-encoded genes. A-site transformed reads were summed across the first four codons with signal for each gene and normalized by the total number of such reads. For genes with 5′ UTRs (*MT-ATP6*, *MT-ND4*, *MT-CO3*, *MT-ND5-dORF*), this includes codons 2–5, where codon 5 is a stop codon for *MT-ND5-dORF*. For genes with a very short or no leader (all others), this includes codons 6–9. For *MT-ATP6*, the signal is confounded by reads from *MT-ATP8*, which overlaps the first 15 codons. Samples are plotted in the order listed and error bars show range across replicates. **E** Variants at *MT-ND5-dORF* ATG (in green), which is antisense to *MT-ND6* (in grey) in the orientation indicated by the schematic. ATA: canonical alternative mitochondrial start codon. GTG: variant start codon shown to efficiently initiate translation. ACG: capable of initiation in reconstituted mammalian mitochondria translation (Lee et al, 2021). AGG: ambiguous (stop or frame-shift inducing). **F** Genome-wide analysis of SNVs at ATGs. Only equivalently oriented ATGs opposite to codons in known ORFs were included to equalize the constraints (see “Methods”). ATGs were required to have at least 2 SNVs to be included

Across all cell lines analyzed, we observed a peak of ribosome density in the *MT-ND5* 3′ UTR. The density starts with high A-site occupancy one codon downstream of an AUG initiation codon (Fig. 2A), characteristic of an initiating ribosome with its P site on the start codon. The AUG begins a short ORF encoding a four-amino-acid putative peptide (Fig. 2B). However, in-frame RPF density continues beyond the stop codon, possibly by the process of stop codon read-through. We observe abundant read-through of *MT-CO1*, *MT-CO2*, and *MT-ND5*, three of the four messages that have true 3′ UTRs (Additional file 1: Figure S2A). At each of these, as well as at the short ORF, the stop codon is followed by a C, a context that promotes read-through in the cytosol [34]. These observations suggest that stop codon read-through may be prevalent in human mitochondria and could indicate the production of a four-amino-acid or longer peptide encoded in the *MT-ND5* 3′ UTR.

To determine whether the *MT-ND5* downstream ORF (*MT-ND5-dORF*) is likely to be translated, we first investigated the distribution of the lengths of reads that map to the ORF. We compared the read length distribution to a region of the same size in a known ORF and across a tRNA (Additional file 1: Figure S2B). The lengths of reads mapping to *MT-ND5-dORF* are not significantly different from those mapping to known coding regions (Kolmorgorov-Smirnov test, *p*=0.136) but are significantly different from those mapping across tRNA (Kolmorgorov-Smirnov test, *p*=0.000279), suggesting that they are derived from ribosome protection and not protection from another RNA-binding protein or structured/double-stranded RNA. Next, we devised a strategy to score all start codons across the genome using mitoribosome occupancy. Three features were integrated into the scoring design: (1) P-site pausing, (2) periodicity/in-frame signal, and (3) overall ribosome occupancy before and after the start codon (see “Methods” for details; Additional file 1: Figure S2C). Start codons of known genes all scored highly, regardless of whether the ORF they began had no leader, a short leader, or a long 5′ UTR as in overlapping ORFs (Fig. 2C, Additional file 1: Figure S2D). *MT-ND5-dORF* scored amongst the known genes across cell lines (Fig. 2C, Additional file 1: Figure S2D), suggesting that it might be translated. Mitoribosome density across *MT-ND5-dORF* in different cell lines indicates that it is engaged at levels comparable to mtDNA-encoded OXPHOS Complex I subunits (Fig. 2D).

We also investigated whether *MT-ND5-dORF* resists accumulating mutations. We analyzed a catalog of mtDNA variants across a population of nearly 200,000 individuals not enriched for mitochondrial disorders [35]. Of 255 single-nucleotide variants (SNVs) of the *MT-ND5-dORF* AUG start codon, 219 produce a known alternative mitochondrial start codon (Fig. 2E). Of the 36 that do not, 35 result in ACG, a codon supporting low levels of translation initiation by mitoribosomes in vitro [36]. We compared the *MT-ND5-dORF* AUG codon to other mtDNA AUG codons. In particular, we investigated those that are antisense to codons in known ORFs in an equivalent manner (i.e., those antisense to the second and third position of a codon and the first position of the subsequent codon). A large majority (82%) of these similarly situated AUG codons had a smaller fraction of conservative changes across individuals (Fig. 2F). Consequently, the *MT-ND5-dORF* start codon is retained in the human population, suggesting a possible functional role for *MT-ND5-dORF* translation.

### Balanced mitochondrial and cytosolic translation across cell types

**Fig. 3:**
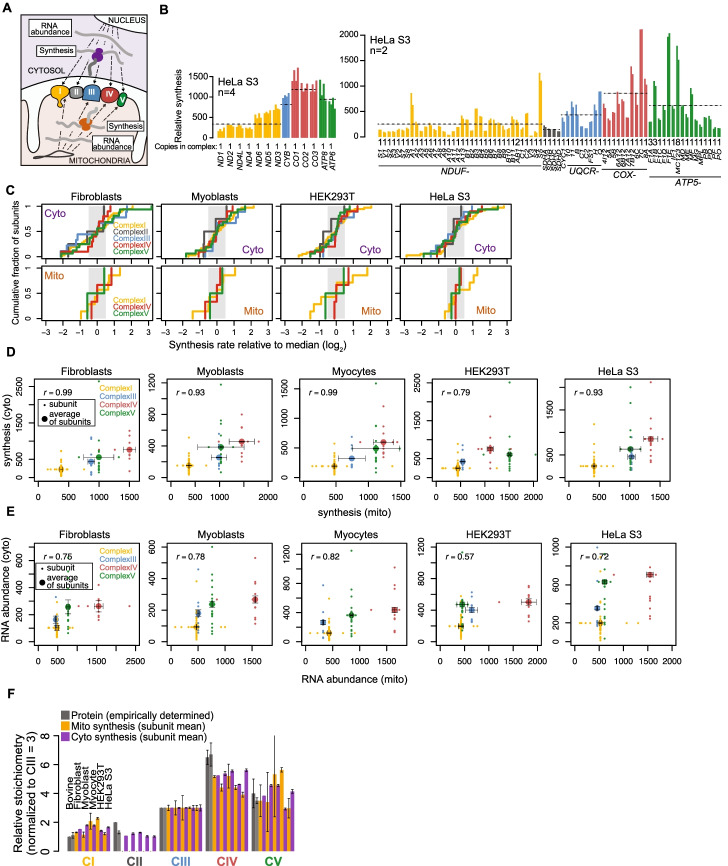
Balanced translatomes exist in five human cell lines. **A** Flow of genetic information to encode dual-origin OXPHOS complexes in human cells. **B** Relative synthesis values of mtDNA- (left graph) and nDNA-encoded (right graph) OXPHOS subunits. Cytoribosome profiling data are from Wu et al. [42]. Each replicate is shown as an individual bar, values given in Additional file 3: Table S2. Dotted lines show averages across all subunits and samples. Numbers below bars show stoichiometry of each subunit. Synthesis units are tpm/100 for mitochondrial synthesis and tpm for cytosolic synthesis (note that mitochondrial and cytosolic synthesis absolute values cannot be compared across compartments). **C** Cumulative distribution of relative synthesis rates for nDNA-encoded subunits (top, Additional file 3: Table S2 and “Methods” for details) and mtDNA-encoded subunits (bottom) of the dual-origin OXPHOS complexes. Log_2_-fold differences in synthesis levels compared to the median value for each complex are shown. Shaded region indicates 2-fold spread. **D** Synthesis of mtDNA-encoded subunits (tpm/100) compared to synthesis of nDNA-encoded core subunits (tpm) for each complex. Small dots show individual subunits, large dots show average synthesis of subunits within each corresponding complex, following the color code displayed. Fibroblast and HeLa S3 cytoribosome profiling data are from Tirosh et al. [43] and Wu et al. [42], respectively. Myocytes are post-differentiation-induction day 2 myoblasts. Individual subunit synthesis for each replicate is shown in Fig. 3B (HeLa S3), and Additional file 1: Figure S3A. Error bars show range in averages across replicates. **E** RNA abundance of mtDNA-encoded subunits (tpm/100) compared to RNA abundance of nDNA-encoded subunits (tpm) for each complex. Subunits included are identical to those in **D**. Small dots show individual subunit RNA abundance, large dots show average RNA abundance of subunits within each corresponding complex, following the color code displayed. Error bars show range in averages across replicates. All panels show Pearson correlation, *r*. **F** Stoichiometry of average synthesis values for each complex relative to Complex III, which is set to 3, following published convention. Dark gray and light gray bars show empirically determined complex stoichiometry as determined in [1, 2], respectively

Across bacteria and eukaryotes, subunits of most multiprotein complexes are synthesized proportionally to their final complex stoichiometry, with synthesis levels falling within a 2-fold range [37–39]. Nuclear DNA-encoded mitochondrial complex subunits are a noted exception [37]. Interestingly, our mitoribosome profiling results showed that the mitochondrial-encoded subunits are also an exception as they are not synthesized proportionally to their complex stoichiometry (Fig. 3B, Additional file 1: Figure S3A). We observed wide-ranging synthesis levels across the mtDNA- and nDNA-encoded subunits for each of the four dual-origin OXPHOS complexes (Fig. 3A,C, shaded region shows 2-fold range). We were unable to make any connection between subunit synthesis levels and their assembly order [40] or protein turnover rates [41]. However, we did notice similar patterns of average complex synthesis between mitochondria and cytosol. Complex I appears lowest when all subunits are considered, sequentially followed by Complex III, Complex IV, and Complex V (Fig. 3B, Additional file 1: S3A, Additional file 3: Table S2). Indeed, when comparing the average synthesis for all subunits in the complex, OXPHOS synthesis levels in the cytosol and mitochondria were highly correlated in all cell lines studied (Fig. 3D). This correlation does not hold when considering the least abundant component of each complex, which intuitively would be expected to be rate limiting for complex assembly (see “Discussion”).

As synthesis levels are a product of both RNA abundances and translation rates, we investigated whether the correlated synthesis across compartments was driven by RNA levels. We found that RNA abundances were also positively correlated, but to a lesser extent (Fig. 3E), revealing the role of translation regulation in tuning synthesis levels to promote balanced synthesis of OXPHOS subunits across compartments in human cells. To determine whether translatome balance is observed in other eukaryotes, we re-analyzed our published mitochondrial and cytosolic ribosome profiling data from *S. cerevisiae* [12]. Despite the synchronous translational programs during mitochondrial biogenesis [12], we found no correlation between mitochondrial and cytosolic average complex synthesis in yeast (Additional file 1: Figure S3B). These results underscore the distinct regulatory mechanisms acting to ensure mitonuclear balance in producing and maintaining OXPHOS complexes.

What do the averages of complex subunit synthesis levels mean biologically and why are they balanced across compartments? The steady-state abundances of the OXPHOS complexes with respect to each other are known for bovine mitochondria [1, 2] and are consistent with the averages of synthesis levels we measured for each complex (Fig. 3F). We propose that the average synthesis in both compartments is tuned such that the complexes are synthesized proportionally to each other, in sharp contrast to the lack of proportional synthesis for subunits within each complex. The final fine-tuning of individual subunit abundance might then be achieved by posttranslational mechanisms.

### Imbalanced translatomes induce proteostasis stress response pathways

**Fig. 4:**
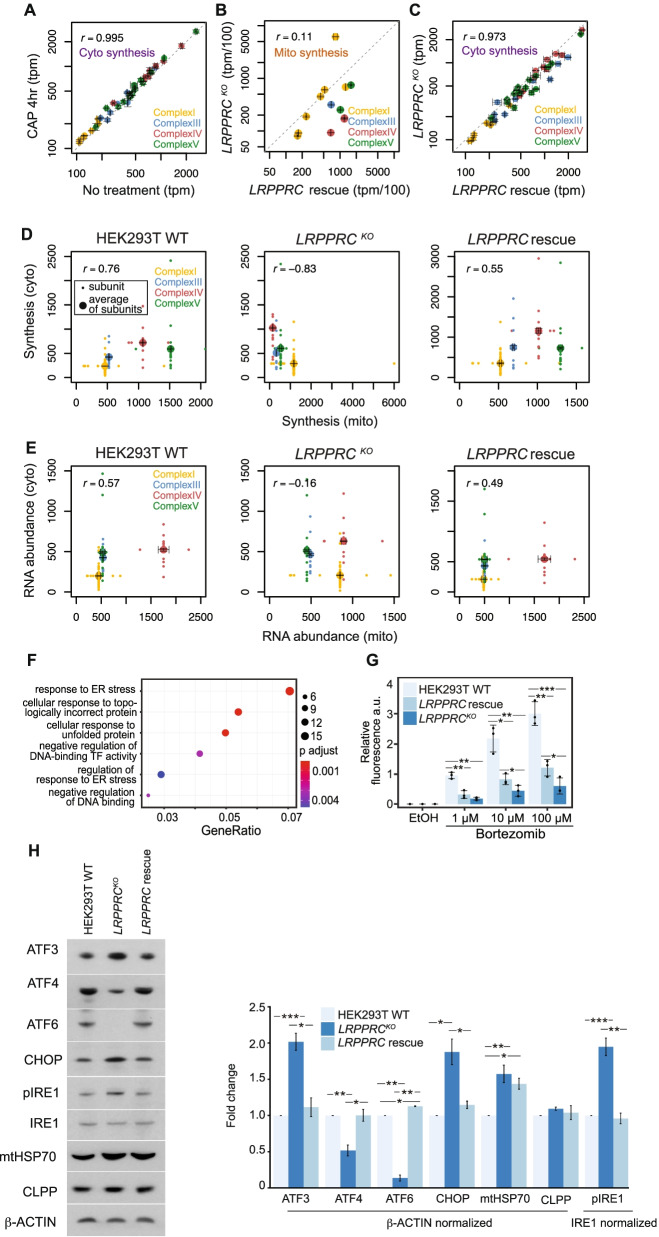
Lack of feedback between translatomes results in their imbalance and leads to the activation of proteostasis genes. **A** Comparison of relative synthesis levels for cytosolic-translated OXPHOS subunits with and without 4 h of chloramphenicol (CAP) treatment (100 μg/mL) to inhibit mitochondrial translation. **B** Relative synthesis for mitochondrial-encoded OXPHOS subunits in *LRPPRC*^KO^ cells compared to a reconstituted cell line (*LRPPRC* rescue), expressing LRPPRC in the *LRPPRC*^KO^ background. **C** Relative synthesis comparison as in (B) but for nuclear-encoded OXPHOS subunits. **D** Average synthesis of mtDNA-encoded subunits (tpm/100) compared to average synthesis of nDNA-encoded core subunits (tpm) for each complex in wild-type HEK293T cells, *LRPPRC*^KO^ cells, and *LRPPRC* rescue cells. **E** Average RNA abundance of mtDNA-encoded subunits (tpm/100) compared to average RNA abundance of nDNA-encoded core subunits (tpm) for each complex in *LRPPRC* cell lines. Error bars throughout show range in averages across replicates, and all panels show Pearson correlation, *r*. **F** GO term enrichment analysis of significantly (adjusted *p*-value < 0.05) differentially expressed genes that show a minimum twofold increase in the *LRPPRC*^KO^ cells compared to the *LRPPRC* rescue cell line. Shown are the GO terms after filtering for redundancy. The size of points represents the number of genes in each GO term. *p* adjust = adjusted *p*-value. TF = transcription factor. **G** Cell toxicity graph after 72-h bortezomib treatment. HEK293T WT, *LRPPRC*^KO^, or *LRPPRC* rescue cells were treated with increasing amounts of bortezomib or the vehicle ethanol (EtOH). Cell death was measured via a fluorescence dye and living cells by indirect measurement of ATP levels via luminescence. Shown are the mean relative fluorescence arbitrary units (a.u.) normalized to the respective luminescence of 3 technical replicates, with WT ethanol treated having 2 technical replicates. The range bars represent the standard deviation. The stars represent significant *p*-values, determined with a Welch two-sample one-sided (less) *t* test, * signifies *p*-value < 0.05, ** signifies *p*-value < 0.01 and *** signifies *p*-value < 0.001. Two other biological replicates are shown in Additional file 1: Figure S4L. **H** Left panel shows the immunoblot analysis using whole cell lysates from HEK29*3* WT, *LRPPRC*^KO^, or *LRPPRC* rescue cells probed for antibodies of markers of the ER and mitochondrial UPR pathways. Bar graphs show signals normalized by β-ACTIN values and compared to WT to determine fold changes. IRE1 levels are shown as ratios of its phosphorylated form (pIRE1) compared to total (IRE1). Error bars indicate standard deviation between two replicates. *T* test was used to test for significant changes. * signifies *p*-value < 0.05, ** signifies *p*-value < 0.01, and *** signifies *p*-value < 0.001

In yeast, cytosolic translation controls mitochondrial translation, directly facilitating balanced mitochondrial and cytosolic protein synthesis [12, 13]. We asked whether cytosolic translation also controls mitochondrial translation in human cells in order to generate the correlated translatomes we observed. Inhibition of cytosolic translation for 30 min or 2 h had no effect on relative or absolute mitochondrial protein synthesis (Additional file 1: Figure S4A,B,C). Similarly, inhibiting mitochondrial translation did not affect cytosolic translation in the short term (Fig. 4A; Additional file 1: Figure S4D,E,F). Thus, in contrast to the situation in yeast, we did not observe rapid communication from cytosolic to mitochondrial translation programs.

To test the impact of chronic mitochondrial synthesis deficiency on cytosolic translation, we targeted LRPPRC, an RNA-binding protein that regulates mitochondrial mRNA stability and interacts with the mitoribosome [21–25]. We used CRISPR/Cas9 editing to generate single-cell clones in which expression of *LRPPRC* is ablated (Additional file 1: Figure S4G,H,I), leading to extremely poor respiratory competence (~10% of WT cells; Additional file 1: Figure S4J). Mitochondrial protein synthesis was dramatically depleted in *LRPPRC*^KO^ cells compared to a cell line that overexpressed LRPPRC in the *LRPPRC*^KO^ background (*LRPPRC* rescue) (Fig. 4B). Despite the attenuation in the synthesis of most mitochondrial transcripts (Fig. 4B), cytosolic protein synthesis of OXPHOS nuclear-encoded subunits was barely affected (Fig. 4C). Mitochondrial and cytosolic protein synthesis became uncorrelated in the *LRPPRC*^KO^ cells (Pearson’s *r* = −0.83) (Fig. 4D). RNA expression was also imbalanced, but not to the same degree (Pearson’s *r* = −0.16) (Fig. 4E). The reconstituted cell line restored the positively correlated mitochondrial and cytosolic translatomes observed in wild-type cells (Fig. 4D, E). These results are consistent with the imbalanced steady-state levels of OXPHOS transcript and protein levels in mice hearts after loss of LRPPRC and other factors impacting mtDNA expression [44]. Thus, when mitochondrial gene expression is constitutively impaired, a compensatory reduction in nuclear OXPHOS expression does not occur, suggesting that these cells lack active feedback mechanisms to maintain mitonuclear translational balance.

We asked whether a loss of balanced translatomes is associated with a stress response, perhaps due to a loss of cellular proteostasis. Gene set enrichment analysis of *LRPPRC*^*KO*^ RNA-seq data revealed the upregulation of genes involved in protein quality control (Fig. 4F, Additional file 1: Figure S4K, Additional file 4: Table S3, Additional file 5: Table S4). These transcriptional changes are consistent with the activation of protein quality control pathways after *LRPPRC* silencing and when nuclear DNA-encoded mitochondrial proteins are in excess [45, 46]. Western blot analysis of *LRPPRC*^*KO*^ cell lysate showed an increase in the levels of CHOP, ATF3 and phosphorylated IRE1, accompanied by a decrease in ATF6 and ATF4, suggesting a complex adaptive response to protein folding stress exists in these cells. Levels of mtHSP70 were increased, indicating that mitochondrial stress was targeted. Additionally, *LRPPRC*^*KO*^ cells were less sensitive to bortezomib, a proteasome inhibitor, consistent with the higher synthesis of proteasomal subunits in these cells (Additional file 1: Figure S4L). We have interpreted these results as indicating that the induction of the protein quality control genes may offer protection from further proteotoxicity [47] (Fig. 4G, Additional file 1: Figure S4M). In sum, loss of LRPPRC disrupts mitonuclear translational balance and promotes the upregulation of proteostasis pathways.

## Discussion

A fundamental question in mitochondrial biology and cellular proteostasis is how the mitochondrial and nuclear genomes produce OXPHOS complexes in a coordinated fashion despite involving disparate gene expression systems operating in separate cellular compartments. In human cells, protein quality control pathways rapidly degrade orphaned OXPHOS proteins [6, 11, 48], and nDNA-encoded subunits of each OXPHOS complex are not synthesized in stoichiometric balance [37] (Fig. 3B,C; Additional file 1: Figure S3A). These observations led to proposals that human cells may not need to coordinate cytosolic and mitochondrial protein synthesis [6, 49]. In this study, we asked whether nuclear and mitochondrial OXPHOS genes are co-expressed in human cells.

To quantitatively capture mitochondrial translation, we tailored ribosome profiling for the mitoribosome, resolving unappreciated features of human mitochondrial protein synthesis. We resolved mitoribosomes initiating on mt-mRNAs with short or absent 5′ leaders and observed slow elongation until the mRNA channel was fully occupied. The high sensitivity of our approach uncovered *MT-ND5-dORF*, an ORF in the 3′ UTR of *MT-ND5* that may be translated. The start codon of *MT-ND5-dORF* is retained in the human population and we observed mitoribosome density across the ORF at comparable levels to densities found on other OXPHOS ORFs. An abundance of functional small peptides are encoded on the nuclear genome and in bacterial genomes [50, 51]; however, as the mitochondrial genome remains largely refractory to genome editing, it will be challenging to determine the functional impact of *MT-ND5-dORF*.

The proportional synthesis of subunits within complexes is a phenomenon conserved from bacteria to mammals and has presumably evolved for efficient resource allocation [37, 38]. The dual-origin OXPHOS complexes are an exception [37, 38], but here we show that, overall, the “correct” number of ribosomes is allocated to each complex when considered as a whole. Instead of each subunit getting an equal share, some get more and some get less, with differential protein turnover having a more prominent role in reaching the final subunit stoichiometry in these complexes (also see [39]). Perhaps this is due to regulatory mechanisms that aid in the assembly of these dual-origin complexes. Remarkably, when the OXPHOS complexes are considered as a group, we see that ribosome allocation is honed after all to match the relative abundances of each complex. Furthermore, this ribosome allocation is balanced in both the mitochondrial and cytosolic compartments. Mitochondrial and nuclear transcriptional programs produce OXPHOS RNA abundances that correspond modestly across complexes when averaged across each complex. Then ribosome allocation sharpens the correspondence so that the average synthesis levels of the components of each complex are nearly perfectly correlated across compartments.

Synthesis levels of the least abundant (potentially rate-limiting) subunit in each complex are not correlated across compartments. OXPHOS complexes display very large differences in half-lives indicating that subunits within already formed and functional complexes are frequently replaced [41]. Thus, many highly produced subunits could be going into pre-existing complexes rather than being part of de novo complex assembly, resulting in the least produced subunit for each complex not being rate limiting for complex assembly.

In yeast, translation also plays a significant role in mitonuclear coregulation of OXPHOS subunits [12]. However, in that system, synchronous mitochondrial–cytosolic translation programs depend on cytosolic translation, indicating that direct communication between the translation systems enables the coregulation. By contrast, in human cells, mitochondrial synthesis levels do not require ongoing cytosolic translation, at least on the time scale of hours. These differences are consistent with the paucity of mitochondrial translation regulators and UTRs that may represent a lack of direct control mechanisms for human mitochondrial translation. So how are mitochondrial and cytosolic translation fluxes maintained so that they correspond so precisely? We propose that on longer time scales, human mitochondrial translation adapts to the influx of nuclear DNA-encoded OXPHOS proteins [52], which requires the orchestrated effort of many mitochondrial gene expression regulators, including LRPPRC. Finally, precisely balanced translatomes are likely a continual challenge for the cell to maintain. Yet the consequence of imbalance is high: proteostasis collapse. Thus, we anticipate that the tight reciprocity of mitochondrial and cytosolic translatomes represents crucial vulnerabilities in cellular proteostasis and mitochondrial function.

## Conclusion

Prediction of the novel *MT-ND5-dORF* showcases the impact of precision mitoribosome footprinting, which provides high coverage and subcodon resolution for determination of reading frame. Our optimized approach opens the door for detailed studies of the effects of perturbation, disease, and therapeutics. Furthermore, precise quantification of OXPHOS subunit synthesis levels revealed that OXPHOS complexes are synthesized proportionally across compartments and proportionally to their relative final abundances. This shows proportional synthesis on a higher order in contrast to that of subunits within a complex. Although the balanced mitochondrial and cytosolic synthesis does not rely on rapid feedback between the two translation systems, its loss results in the induction of proteotoxicity pathways, underscoring the need for coordination between expression of the cell’s genomes.

## Methods

### Cell cultures

Dermal human fibroblasts, HeLa S3, Mouse NIH 3T3, and HEK293T were grown in DMEM (Thermo, CAT 11965092), supplemented with 10% FBS (Thermo Fisher, CAT A3160402). Human myoblasts and myocytes were grown in the Human Skeletal Muscle Basal Media Kit (Cell Applications, CAT 151K-500). Unless otherwise indicated, all cell cultures were grown in 15 cm dishes at 37 °C, 5% CO_2_, in the absence of antibiotics to 75–80% confluency. Dermal human fibroblasts and myoblasts were from anonymous healthy control samples kindly provided by Dr. Brendan Battersby (Institute of Biotechnology, University of Helsinki.)

### Myoblast differentiation to myocytes

Human myoblasts were cultured in Human Skeletal Muscle Basal Media Kit (Cell Applications, CAT 151K-500) to 80% confluency. Differentiation was induced by shifting cell cultures to rich medium (DMEM, Thermo Fisher Scientific, CAT 11965092) supplemented with 2% horse serum (Thermo Fisher Scientific, CAT 26050070) and 0.4 μg/mL dexamethasone (Sigma, CAT D4902). Myocytes were harvested 2 days post-differentiation-induction.

### Drug treatments

Cells exposed to ribosome inhibitors were grown to 70% confluency, as explained above, before addition of the drug. Growth medium was replaced by a medium containing 100 μg/mL of the inhibitor [cycloheximide (Sigma, CAT C769), chloramphenicol (Sigma, CAT C3175), or anisomycin (Sigma, CAT A9789)], and 100 μg/mL of uridine (Sigma, CAT U3750). Treatments were done under the same incubation conditions as growth, for the indicated times.

### Generation of mutant cell lines

To create the *LRPPRC* knockout, we first obtained a pool of HEK293T cells that had been transfected with the guide RNA 5′-GAGGACUACUGAGCCCAGCC-3′ targeting *LRPPRC* exon 2, from Synthego (Menlo Park, CA). This pool was then plated into 96 well plates to screen for individual clones and these clones were screened for presence of LRPPRC by western blot (Santa Cruz, sc-166178). Clones that showed absence of LRPPRC by western blot were then genotyped by subcloning the edited section into the pCR 2.1-TOPO TA vector (Thermo Fisher CAT:451641). Clones were then picked and the plasmid was sequenced by Sanger sequencing (GeneWiz). To create the reconstituted cell line, a Myc-DDK tagged *LRPPRC* ORF plasmid was obtained from OriGene (CAT: RC216747). This ORF was then subcloned into a hygromycin resistance-containing pCMV6 entry vector (OriGene, CAT PS100024). *LRPPRC*^*KO*^ cells were then transfected with 1.5 μg of plasmid using endofectin (GeneCopoeia, CAT EF014) as the transfection agent. They were then selected for 2 weeks on 200 μg/mL hygromycin (Invitrogen, CAT 10687010), and LRPPRC levels were checked by western blot. For experiments conducted with the *LRPPRC*^*KO*^, and control cell lines (WT HEK293T and reconstituted *LRPPRC*^*KO*^ +WT rescue), we cultured cells in DMEM (Thermo Fisher Scientific, CAT 11965092), supplemented with 10% FBS (Thermo Fisher, CAT A3160402), 100 μg/mL of uridine (Sigma, CAT U3750), 3 mM sodium formate (Sigma CAT 247596), 1 mM sodium pyruvate (Thermo Fisher Scientific, CAT 11360070), and 1 mM GlutaMAX (Thermo Fisher Scientific, CAT 35050061). For the cell toxicity assay, cells were grown either in normal DMEM or DMEM without phenol red and without sodium pyruvate (Thermo Fisher Scientific, CAT 21063029), supplemented with 10% FBS (Thermo Fisher, CAT A3160402), 100 μg/mL of uridine (Sigma, CAT U3750), 3 mM sodium formate (Sigma CAT 247596), 2 mM sodium pyruvate (Thermo Fisher Scientific, CAT 11360070), and 1 mM GlutaMAX (Thermo Fisher Scientific, CAT 35050061).

### Polarographic measurements in mutant cell lines

Endogenous cell respiration was measured polarographically at 37 °C using a Clark-type electrode from Hansatech Instruments (Norfolk, UK). Briefly, trypsinized cells were washed with permeabilized-cell respiration buffer (PRB) containing 0.3 M mannitol, 10 mM KCl, 5 mM MgCl_2_, 0.5 mM EDTA, 0.5 mM EGTA, 1 mg/ml BSA, and 10 mM KH_3_PO_4_ (pH 7.4). The cells were resuspended at approximately 2 × 10^6^ cells/ml in 0.5 mL of the same buffer air-equilibrated at 37 °C. The cell suspension was immediately placed into the polarographic chamber to measure endogenous respiration. Subsequently, complex IV activity was inhibited using 0.8 mM KCN to assess the mitochondrial specificity of the oxygen consumption measured. Values were normalized by total cell number.

### Cell toxicity assay

To test the toxicity of Bortezomib (Cayman Chemical Company, CAT 10008822), WT HEK293T, *LRPPRC*^KO^ HEK293T, or *LRPPRC*^KO^ rescue cells were grown in a black-walled 96-well plate (Corning Incorporated costar®, CAT CLS3603-48EA). The same growth media with or without phenol red was used as described in the mutant generation section. In total, 5000 cells per well were seeded in a 50-μl volume and left to adhere for 1 to 2 h. The adherence of the cells was checked by visual inspection by light microscopy. Media supplemented with increasing amounts of the protease inhibitor bortezomib (final concentrations 1 μM, 10 μM, and 100 μM) were added to the well. The vehicle ethanol (EtOH) was used as a control (final concentration 1% (v/v)). Cells were grown for 72 h. The toxicity of the drug was measured using the CellTox™ Green Cytotoxicity Assay from Promega (CAT G8742) as described in the instructions. In brief, the dye was mixed with the assay buffer to gain a 5× concentrated working solution. The solution was added to a final 1× concentration, and the plate was transferred into a plate reader (Tecan Infinite 200 Pro). To allow for mixing, the plate was incubated with shaking for 60 s at an amplitude of 1 mm, followed by 14 min wait time, and another 60 s of shaking. After 10 s of settling, the fluorescence was measured using a 485-nm emission and 535-nm excitation filter set. Since the cell toxicity measures the amount of accumulated dead cells, the cell number needs to be determined. To this end, the CellTiter-Glo® Luminescent Cell Viability Assay from Promega (CAT G7571) was used to measure ATP levels for an estimate of living cells. Here, the 2x reagent was added to the before mix and the plate was transferred into the plate reader. To mix the reagent with the media, the plate was incubated with shaking for 120 s at 1 mm amplitude followed by a 10 min incubation without shaking. Afterwards, luminescence was measured. For analysis, first the background was subtracted from raw fluorescence or raw luminescence values. The fluorescence of each well was normalized to the luminescence measured of the respective well. Each biological replicate (*n*=3) includes two to three technical replicates. Analysis was performed in R. For each biological replicate, the measurements for the *LRPPRC*^KO^, WT, and *LRPPRC*^KO^ rescue cells were compared using the Welch two-sample one-sided (“less”) *t*-test in R, an example code: t.test (KO100R1, WT100R1, alternative = "less").

### General nucleic acid and protein methods

Northern blots were performed to assess mitoribosome footprint size. We isolated RNA from mitomonosome-enriched sucrose gradient fractions by extracting with 1:1 volume of acid-phenol/chloroform (Thermo Fisher Scientific). Purified RNA was separated on a 15% polyacrylamide TBE-urea gel. After transfer to a nylon membrane (Hybond N+), blots were probed at room temperature with ^32^P-dATP-internally labeled (Perkin Elmer) random hexamer-primed DNA fragments synthesized from 400 to 500-bp PCR-generated templates for *MT-CO2*. Blots were exposed to a storage phosphor screen and visualized using a Typhoon instrument. Proteins were resolved before western blotting on NuPAGE Novex Bis-Tris gels (Thermo Fisher Scientific). After transfer to a nitrocellulose membrane, blots were probed for primary antibodies against mitoribosome subunits MRPL12 (Proteintech, 14795-1-AP) and MRPS18-B (Proteintech, 16139-1-AP). Other antibodies used were as follows: COI (ABCAM, ab14705), HSP60 (Cell Signaling, 4870S), LRPPRC (Proteintech, 21175-1-AP), ATF3 (Cell Signaling, 33593S), ATF4 (Cell Signaling, 97038S), ATF6 (Cell Signaling, 65880T), CHOP (Proteintech, 66741-1-Ig), IRE (Proteintech, 27528-1-AP), IRE1 alpha [p Ser724] (Novus, NB100-2323SS), mtHSP70 (Thermo Fisher Scientific, MA3-028), CLPP (Proteintech, 15698-1-AP), and b-ACTIN (Cell Signaling, 4967S). Fluorescently labeled secondary antibodies (IRDye, LI-COR) were detected using a LI-COR Odyssey instrument.

### In vivo labeling of translation products

Cells were grown in FBS-supplemented complete DMEM, as explained in the cell culture section above, to 80% confluency. To equilibrate cells to the labeling conditions, growth medium was replaced with labeling medium (DMEM depleted of cysteine, Thermo Fisher Scientific CAT 21013-024) 30 min before beginning of labeling. After 30 min of incubation at 37 °C, 5 % CO_2_, the corresponding cytosolic or mitochondrial translation inhibitor was added to the plates to a final concentration of 100 μg/ml. Treatment was performed for the indicated times. After treatment completion, 200 μCi/mL of EasyTag labeling mix (35S-cysteine/35S-methionine, Perkin Elmer CAT NEG772007MC) was added to each plate. Labeling was followed for the indicated times. To collect, cells were rinsed twice with cold PBS 1× pH:7.4, in 1 mL of 1× PBS pH:7.4, scraped using a cell lifter and pelleted by centrifugation at 1500*g* for 10 min at 4 °C. To prepare samples for electrophoresis, cell pellets were extracted with 1× gel loading buffer (95 mM Tris–HCl, pH:6.8, 7.5% glycerol, 2% SDS, 0.5 mg/ml bromophenol blue, 50 mM DTT). Samples were separated in a 17.5% acrylamide gel (20 cm). After transferring to nitrocellulose membrane, signals were collected by a phosphor imager screen and visualized using a Typhoon instrument.

### Mitoribosome profiling

Cell cultures of biological replicates were grown independently in 15-cm dishes to 70–80% confluence. Medium was aspirated and cells were quickly rinsed once with ice-cold 1× PBS pH 7.4. Lysis was performed on ice in a glass homogenizer with a loose setting. We used 50 μL of lysis buffer for 10^6^ cells, for example, 500 μL to lyse a 15-cm dish of human fibroblasts and 1000 μL to lyse a 15-cm dish of HeLa S3 cells. Cells subjected to drug treatments were lysed in the presence of the indicated drug. Lysis buffer composition was carefully designed to preserve the integrity of the mitoribosome. We used lauryl maltoside at 0.25% to solubilize mitochondrial membranes, while preserving their protein complex integrity, in combination with 50 mM NH_4_Cl, 20 mM MgCl_2_, 0.5 mM DTT, 10 mM Tris, pH 7.5, and 1× EDTA-free protease inhibitor cocktail (Roche). To normalize for global changes in mitochondrial translation, we introduced a mouse spike-in control. Before nuclease digestion, mouse (NIH 3T3) lysate was mixed in with the human at 95%:5% (human:mouse) OD_260_ equivalent volumes. Mitoribosome footprints were generated using 8 units/μL of RNase If (NEB), digesting 450 μL of whole cell lysate at room temperature for 30 min, without rotation. Digestion was stopped by adding 80 units of SUPERaseIn (Thermo Fisher Scientific). To isolate mitoribosomes, lysates were clarified at 10,000 RPM for 5 min at 4 °C, loaded on 10–50% linear sucrose gradients and centrifuged in a Beckman ultra-centrifuge at 40,000 RPM for 3 h at 4 °C using a SW41Ti rotor. Gradients were mixed and fractionated using a BioComp instrument. The mitomonosome fraction was identified using western blots for MRPL12 and MRPS18B (see “General nucleic acid and protein methods” above). Mitoribosome footprints were recovered by 1:1 volume phenol/chloroform extraction of the monosome fraction. RNA was separated in 15% polyacrylamide TBE-urea gels, excising sizes between 28 and 40 nucleotides. Libraries were prepared as described [53], with a few modifications: (1) We did not rRNA deplete and (2) we introduced 10 or 6 randomized nucleotides to the linker ligation at 3′ (/5rApp/NNNNNNNNNNCTGTAGGCACCATCAAT/3ddC/ or /5rApp/NNNNNNCTGTAGGCACCATCAAT/3ddC/) and in many samples also at the reverse transcription step at 5′ (/5Phos/NNNNGATCGTCGGACTGTAGAACTCTGAACCTGTC/iSp18/CACTCA/iSp18/CAAGCAGAAGACGGCATACGAGATATTGATGGTGCCTACAG). Sequencing was performed in an Illumina based Next-seq 500 instrument at the Bauer sequencing facility (at Harvard University).

### Mitoribosome immunoprecipitation

The mitoribosome was immunoprecipitated from the monosome-enriched sucrose fraction using a MPL12 antibody (Proteintech) conjugated to a slurry of DynaBeads protein A from Thermo Fisher Scientific. Mitoribosomes were recovered by eluting beads with mitoribosome profiling lysis buffer without lauryl maltoside but containing 0.1% SDS. Eluted mitoribosome footprints were extracted with 1:1 volume of phenol/chloroform. Libraries were prepared and sequenced as described in the “Mitoribosome profiling” section above.

### Cytoribosome profiling

Cell culture growth and lysis were performed as for mitoribosome profiling (see “Mitoribosome profiling” section above). We used lysis and buffer conditions previously described [54] for polysome profiling. Cytoribosome footprints were generated using 8 × 10^−4^ units/μL of RNase I (Epicentre), digesting 450 μL of lysate at room temperature for 30 min, without rotation. Digestion was stopped by adding 80 units of SUPERaseIn (Thermo Fisher). Isolation of the cytomonosome was performed using the centrifugation and fractionation conditions described for mitoribosome profiling but limiting the centrifugation time to two and a half hours. The cytomonosome fraction was detected by their UV absorbance at 254 nm. Cytoribosome footprints recovery and gel size purification were performed as in mitoribosome profiling but restricting the excision size to 27–33 nucleotides. Library preparation and sequencing techniques were identical to those used for mitoribosome profiling.

### RNA-seq

Some of the lysates prepared for mitoribosome profiling were reserved for RNA-seq. Lysates were further treated with 90 μg/mL of Proteinase K and 0.5% SDS at 42 °C for 20 min. Total RNA was extracted using 1:1 volume of phenol/chloroform. Purified RNA was DNase digested at 37 °C for 30 min with 3 units of RQ1 RNase-free DNase (Promega). We then rRNA-depleted 2 μg of RNA using the RiboMinus Eukaryotic kit v2 from Thermo Fisher (CAT: A15026). RNA was fragmented by alkaline hydrolysis in 5 mM Na_2_CO_3_, 45 mM NaHCO_3_, 1 mM EDTA, pH 9.3 for 25 min at 95 °C. RNA was resolved in 15% TBE-urea gels and size purified including 30–70-nucleotide fragments. Sequencing libraries were prepared and sequenced as above, except for HeLa S3 samples, which were size selected and prepared using the Illumina TruSeq kit.

### Mitoribosome profiling data analysis

Reads were trimmed to remove a ligated 3′ linker (CTGTAGGCACCATCAAT) with Cutadapt [55]. Reads without linker were discarded and the unique molecular identifier (UMI) was then extracted from remaining reads using a custom script but kept associated with each read entry. UMIs consisted of either 6 or 10 random nucleotides at the 3′ end ligated as part of the 3′ linker, and either 0 or 4 random nucleotides at the 5′ end ligated during circularization and originating from the RT primer. Trimmed reads were filtered after alignment using bowtie1 [56] to human rRNA (allowing one internal and one 5′ mismatch) and human tRNA (allowing one internal, one 5′, and three 3′ mismatches). Remaining reads were aligned to the GRCh38 human reference genome merged with the M17 mouse reference genome from GENCODE using STAR 2.7.3 [57] with parameters --outFilterMismatchNoverReadLmax 0.07 and --outFilterMismatchNmax 3. PCR duplicates were identified by their UMI and removed using a custom script. To obtain the spike-in normalization factor, rRNA-filtered reads > 20 nt were mapped to mouse mitochondrial mRNAs using bowtie1 and allowing one 5′ and zero internal mismatches. After removing PCR duplicates, the number of reads mapped was used as the spike-in normalization factor. This strategy results in roughly the fraction of spike-in reads expected from volume:volume fraction of mouse lysate added (5 or 10%), and ≤ 0.1% in samples without added mouse lysate (see Table S1). The offset of the A site from the 3′ end of reads was calibrated using start codons of *MT-ATP6*, *MT-CO3*, and *MT-ND4*, the only CDSs with significant 5′ UTRs. Additionally, periodicity across CDSs was calculated for read 3′ ends for each individual length. By determining the most common subcodon position for 3′ ends of each read size across CDSs, the precise A site offset could be set for each experiment. Offsets from the 3′ end of 30:[−13], 31:[−14], 32:[−15], 33:[−15], 34:[−15 or −16] were typically used. Soft-clipped nucleotides were omitted unless they were 3′ As and thus appear to be part of a poly (A) tail. Shorter read lengths have ambiguous offsets because there is no consistency between whether 5′ or 3′ ends stay constant at a given mitoribosome position and were excluded. A-site transformation was applied to mitochondrial mRNA-mapping reads using a custom script. Mitoribosome profiling datasets for BJ fibroblasts (“Fibro-Rooijers”) [58], additional HEK293T data (“HEK_Pearce”) [59, 60], HCT116 cells (HCT116_Li-1, -2) [61], and RNase footprinting for HeLa (Hela_Jilab-1, -2) [62] were downloaded from GEO (GSE48933, GSE133315, and GSE151987), ArrayExpress (E-MTAB-5519), and the SRA (SRR10491343 and SRR10491342). Relative ribosome occupancies for codons were computed by taking the ratio of the A-site transformed ribosome density in a 3-nt window at the codon to the overall density in the coding sequence.

### Cytoribosome profiling data analysis

Read trimming, filtering, alignment, and PCR duplicate removal was performed identically to mitoribosome profiling data analysis except that filtered reads were aligned to the human genome reference alone without the mouse reference. Cytoribosome profiling datasets for HeLa S3 cells and fibroblasts from [42, 43] were downloaded from GEO (GSE69906 and GSE115162).

### RNA-seq data analysis

Read trimming, filtering, alignment, and PCR duplicate removal was performed identically to mitoribosome profiling data analysis except that default STAR mismatch parameters were used.

### OXPHOS subunit expression analysis

Relative synthesis for OXPHOS genes was calculated as length- and library size-normalized read counts. For mitochondria-encoded OXPHOS subunit relative synthesis, mitoribosome profiling read counts were summed across genes using Rsubread featureCounts [63] with parameters readShiftType = "upstream", readShiftSize = 14, read2pos = 3, allowMultiOverlap = TRUE, useMetaFeatures = TRUE, countMultiMappingReads = TRUE, strandSpecific = 1, GTF.featureType='CDS'. Importantly, the GTF annotation file was modified to exclude the first 6 codons of all genes (which are variable depending on whether there is a 5′ UTR) and all overlapping regions. Multimapping reads are included because nuclear mitochondrial DNA (NUMT) exists in the nuclear genome but does not appear to be the source of reads in mitoribosome profiling libraries as NUMT loci do not have uniquely mapping reads. For mitochondrial genes, relative synthesis is expressed as transcripts per ten thousand (tpm/100): RPK/sum (mitochondrial protein-coding genes RPK) × 10,000. For nuclear DNA-encoded OXPHOS subunit relative synthesis, cytoribosome profiling read counts were summed across protein-coding genes using featureCounts with parameters readShiftType = "downstream", readShiftSize = 17, read2pos = 5, allowMultiOverlap = TRUE, useMetaFeatures = TRUE, countMultiMappingReads = TRUE, strandSpecific = 1, GTF.featureType='CDS'. Multimapping reads are included because several OXPHOS genes have pseudogenes that are not a significant source of uniquely mapping reads. For nuclear DNA-encoded genes, relative synthesis is expressed as transcripts per million (tpm): RPK/sum (all protein-coding genes RPK)×1,000,000. Importantly, several nuclear DNA-encoded OXPHOS subunits have paralogs that encode alternative isoforms. These isoforms do not have significant stretches of nucleotide sequence identity, but they do encode similar proteins that may be interchangeable depending on cell/tissue type. Therefore, isoform tpm counts are summed for the analyses in Fig. 3, Additional file 1: Figure S3, and 4 (e.g., COX4I1 and COX4I2 become COX4I1/2). Of note, relative synthesis values for OXPHOS complex subunits computed with this method are highly correlated (*r*=0.96, data not shown) with relative synthesis values computed from the same data using a masking approach [37] xxx [64], except that here we recover values for seven additional subunits. For both mtDNA- and nDNA-encoded OXPHOS subunit relative RNA abundance, RNA-seq read counts were summed across protein-coding genes using featureCounts with parameters allowMultiOverlap = TRUE, minOverlap = 22, countMultiMappingReads = TRUE, GTF.featureType = 'CDS'. The GTF annotation file was modified to exclude overlapping regions in mitochondrial genes. “CDS” was chosen as the feature to count instead of “exon” for two reasons. (1) UTR annotation is less reliable than CDS annotation and (2) unexpressed isoforms with long UTRs artificially reduce apparent abundance. Library size normalizations were performed separately for mtDNA- and nDNA-encoded subunits resulting in relative abundance values expressed as tpm/100 and tpm, respectively, as described above for ribosome profiling data.

### GO term enrichment analysis of differentially expressed genes

To determine differential expression of the *LRPPRC*^*KO*^ mutant compared to the *LRPPRC* rescue cells, we performed a differential expression analysis using RNA-seq reads counted as described in “OXPHOS subunit expression analysis” above. For differential expression analysis, the R package DESeq2 [65] was used. Default parameters were used, and the counts were normalized using the estimateSizeFactors function. For differential expression results, the method contrast “sampletype” was used. To determine significantly changed genes, genes were filtered for adjusted *p*-value < 0.05. Two sets were generated for GO term enrichment analysis, genes with log2 changes ≥ 1 and with log2 changes ≤ −1. For GO term enrichment analysis, the R package clusterProfiler [66] was used. The unfiltered results table was used to generate the universe gene list used to test against for enrichment. The filtered lists for either significantly up- or downregulated genes were used for testing. GO term analysis was performed using the enrichGO function with the following parameters: keyType = "ENSEMBL", OrgDb = org.Hs.eg.db, ont = "BP", pAdjustMethod = "BH", pvalueCutoff = 0.05, qvalueCutoff = 0.05, readable = TRUE). For Fig. 4F, the resulting GO terms were filtered for redundancy, using the simplify function with the following settings: cutoff = 0.2, by = "p.adjust", select_fun = min, measure = "Wang", semData = NULL. For visualization, the dotplot function was used with showCategory = 10. For Additional file 1: Figure S4K GO terms without filtering were used.

### MT-CO3 initiation analysis

The fraction of processed and unprocessed *MT-ATP8/6-CO3* transcript was calculated from RNA-seq data using a custom python script. Reads with their 5′ end precisely at *MT-CO3* start were counted as coming from processed transcripts, and reads that span the junction were counted as coming from unprocessed transcripts. The fraction of *MT-CO3* translation initiation on processed and unprocessed *MT-ATP8/6-CO3* transcript was calculated from mitoribosome profiling data using a custom R script. Reads with their 5′ end precisely at *MT-CO3* start (vertical arrowhead in Fig. 1E) were counted as coming from initiation on processed *MT-CO3*. Reads that span the junction could result from either *MT-CO3* initiation or *MT-ATP6* termination. Therefore, to count only instances of initiation on unprocessed *MT-CO3*, reads with their 5′ ends highlighted by the diagonal arrowhead in Fig. 1F were summed.

### Ad hoc scoring

Start codons were assigned scores based on mitoribosome profiling data as described in Fig. 2C using a custom R script. Briefly, the genome sequence was split into three frame references and A-site reads falling on the second (middle) position of each were assigned to that reference. Each start and stop codon were also assigned to a reference, as were known genes. Scores were assigned to each AUG, AUU, AUA, and GUG codon in three parts where the final score is the sum of the following: (1) summed fold-change in P-site in-frame reads (inferred from A-site reads one codon downstream) compared to one codon upstream and one codon downstream. (2) Average percent of in-frame reads 20 codons downstream minus average percent of in-frame reads 16 codons upstream. (3) Total number of reads (in all frame references) five codons downstream divided by total number of reads five codons upstream. This final value was log-transformed then doubled to keep its contribution commensurate with the other values. For score frequency histograms shown in Fig. 2C and Additional file 1: Figure S2C, start codons within the reading frame of known ORFs were excluded and rRNA- and tRNA-filtered heavy strand data was used, resulting in no scores for start codons in rRNA or tRNA genes, nor on the light strand (e.g., *MT-ND6*).

### SNV analysis

Alternative start codons resulting from homoplastic variants [35] at ATG codons were counted using a custom R script. Because *MT-ND5-dORF* is encoded by the same DNA (but on the opposite strand) as *MT-ND6*, we limited our analysis to other ATGs in the identical orientation antisense to known genes (see Fig. 2E,F). ATT, ATA, and GTG are counted as alternative start codons, but not ACG, which is capable of translation initiation in vitro [36], but has not been found to be used in vivo in human mitochondria.

## Supplementary Information

Additional file 1: Figure S1. Optimization and adaptability of mitoribosome profiling. Figure S2. Translation of a novel mitochondrial open reading frame. Figure S3. Correlated synthesis of mtDNA- and nDNA-encoded OXPHOS subunits. Figure S4. Translation perturbations.

Additional file 2: Table S1. Library composition and quality characteristics for wild type, untreated samples.

Additional file 3: Table S2. Relative synthesis values for OXPHOS subunits.

Additional file 4: Table S3. Differential expressed (DE) genes in *LRPPRC*^*KO*^*.*

Additional file 5: Table S4. GO terms enriched for differential expressed genes in *LRPPRC*^*KO*^*.*

Additional file 6. Review History.

## Data Availability

*Lead contact* Further information and requests for resources and reagents should be directed to and will be fulfilled by the Lead Contact: L. Stirling Churchman (churchman@genetics.med.harvard.edu) *Materials availability* This study did not generate new unique reagents. **Data and code availability** Raw and processed sequencing data were deposited in the GEO database under the accession number GSE173283 [67]. Scripts and instructions for human mitoribosome profiling data analyses are available at https://github.com/churchmanlab/human-mitoribosome-profiling [68]. We have also deposited our data in Zenodo, under source code: DOI: 10.5281/zenodo.6814723 [69].
