# Supplementary material for: Balanced mitochondrial and cytosolic translatomes underlie the biogenesis of human respiratory complexes
Source: Genome Biol. 2022 Aug 9;23:170. doi: 10.1186/s13059-022-02732-9 (PMC9361522; doi:10.1186/s13059-022-02732-9)
Supplement: Supplementary file 1 — Additional file 1: Figure S1. Optimization and adaptability of mitoribosome profiling. Figure S2. Translation of a novel mitochondrial open reading frame. Figure S3. Correlated synthesis of mtDNA- and nDNA-encoded OXPHOS subunits. Figure S4. Translation perturbations. [file 13059_2022_2732_MOESM1_ESM.docx]

Supplementary Figures for

**Balanced mitochondrial and cytosolic translatomes underlie the biogenesis of human respiratory complexes**

Iliana Soto^1,#^, Mary Couvillion^1,#^, Katja G. Hansen^1^, Erik McShane^1^, J. Conor Moran^2^, Antoni Barrientos^2^, L. Stirling Churchman^1^*

^1^Blavatnik Institute, Department of Genetics, Harvard Medical School, Boston, Massachusetts, 02115 USA

^2^Department of Neurology, University of Miami Miller School of Medicine, Miami, FL 33136 USA

*Corresponding author. Email: churchman@genetics.med.harvard.edu

^#^these authors contributed equally to this work


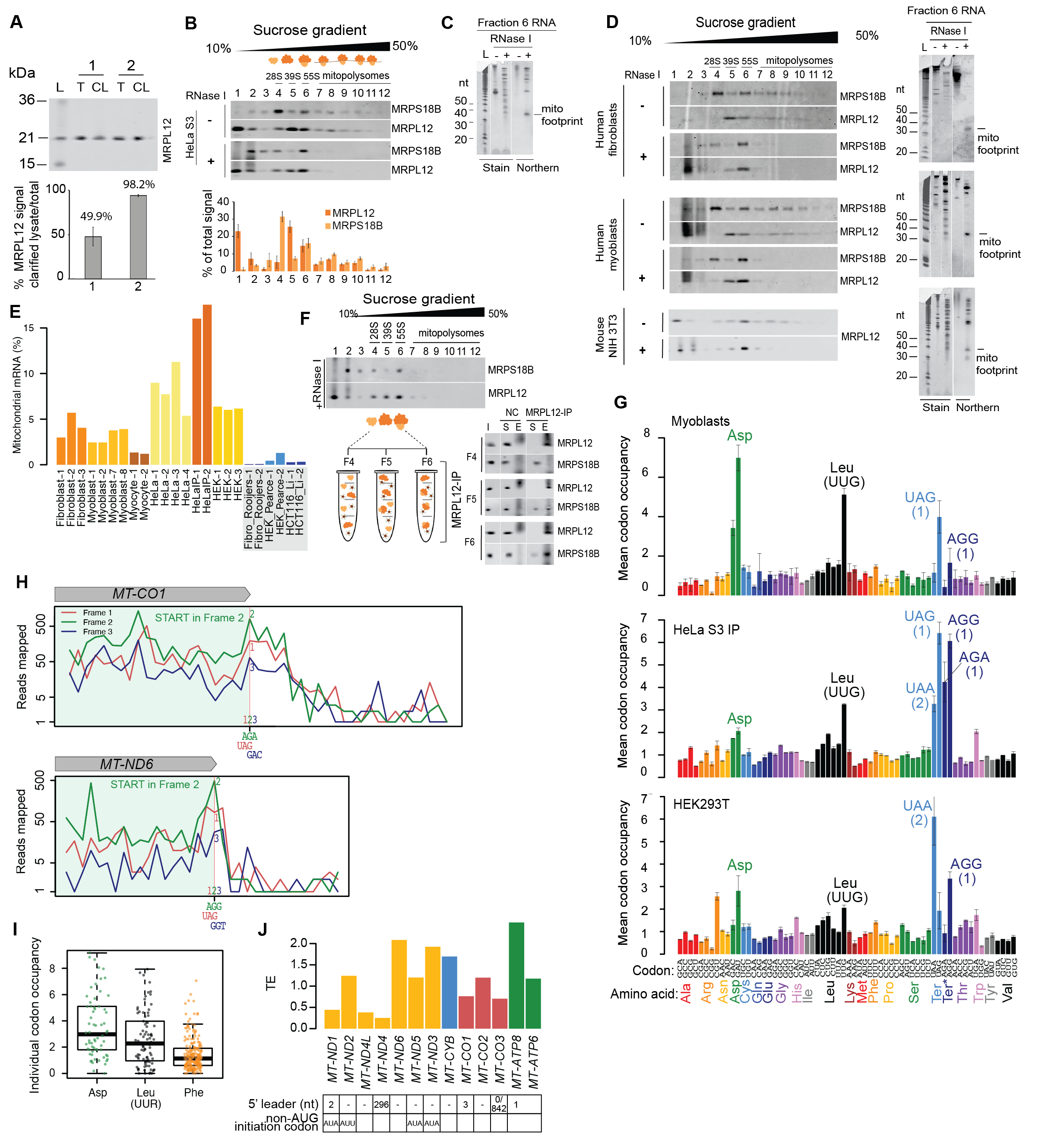


**Figure S1. Optimization and adaptability of mitoribosome profiling.** (**A**) Mitoribosome extraction efficiency after employing previously established (Rooijers et al., 2013) solubilization conditions (1), and our optimized lysis buffer (2). HeLa S3 cells were lysed with either lysis composition, followed by clarification of the lysates. Equal volumes of total (T) and clarified lysates (CL) were subjected to western blots against a mitoribosome subunit antibody (MRPL12). (L) indicates the protein marker used in kilodaltons (kDa). Quantification from two experiments of the total and post-clarification signals revealed the optimized conditions allow for the recovery of >98% of mitoribosomes, compared to only 50% for the existing protocols. Error bars represent the range between two replicates. (**B**) Isolation of mitomonosome (55S) footprints from mitochondrial polysomes. Digested (+RNase I) and undigested (-RNase I) HeLa S3 lysates were clarified and loaded onto linear 10-50% sucrose gradients. Detection of mitoribosomes in each fraction (top panel) was achieved by western blots against proteins of the large (MRPL12) and small subunit (MRPS18B). Signals of both subunits from four experiments were quantified, showing a periodic trend (lower panel). (**C**) Footprints recovered from the mitomonosome fraction (Fraction 6) were subjected to northern blot (*MT-CO2* probe) for assessment of size distribution. (**D**) Mitoribosome isolation conditions were readily adaptable to other cell lines. As shown in **(**B**)** for HeLa S3 cells, digested lysates from human fibroblasts, human myoblasts and mouse NIH3T3 were used to generate and isolate mitoribosome footprints following the sedimentation of the mitomonosome (Fraction 6 RNA) by western blots of large (MRPL12) and small (MRPS18B) mitoribosome subunits (left panels). Footprint size was assessed by northern blot (right panels). (**E**) Percentage of reads in each mitoribosome profiling sample that maps to mitochondrial mRNAs. Bars are colored by cell type with data from this work in yellow/orange and published data in blue. Gray box highlights published wild type, untreated samples from BJ fibroblasts (Rooijers et al., 2013) (Fibro_Rooijers-1, -2), HEK293T cells (Gopalakrishna et al., 2019; Pearce et al., 2017) (HEK_Pearce-1, HEK_Pearce-2), and HCT116 cells (Li et al., 2021) (HCT116_Li-1, -2) analyzed in parallel. Full sample compositions are shown in **Additional File 2: Table S1**. (**F**) Mitoribosome immunoprecipitation. RNase I-digested HeLa S3 lysates were clarified and loaded onto 10-50 % sucrose gradients (left panel). Fractions 4, 5 and 6 were incubated with either MRPL12-conjugated DynaBeads or naked DynaBeads (NC) as negative control. We analyzed subsequent input (I), supernatant (S), and elution products (E) using western blots (right panel), observing the highest amount of the small subunit immunoprecipitated in fraction 6. (**G**) Mitoribosome occupancy across codons. Asp, Leu (UUR), and termination codons are labeled. AGA and AGG Ter* putative termination codons are each present only once (indicated in parentheses), at the ends of *MT-CO1* and *MT*-*ND6*, respectively. R: purine (A or G). Codons with occupancy of >3x expected are labeled. Error bars show range across replicates. (**H**) Stacked frame plot highlighting number of A-site transformed reads in each subcodon position on heavy strand. Vertical red line indicates stop codon with number of reads on in-frame non-canonical AGA/AGG shown by green line, and on -1 frameshifted canonical UAG shown by red line. See example analysis in Figure 3C for how raw A site read counts are transformed to line plots. (**I**) Distribution of occupancies across individual Asp, Leu (UUR) and Phe codons in fibroblasts. Dotted line highlights expected occupancy in the absence of pausing. (**J**) Translation efficiency (TE = relative synthesis values/relative RNA abundance values) of each mitochondrial OXPHOS transcript in fibroblasts. Transcript 5’ end characteristics are listed below plot.


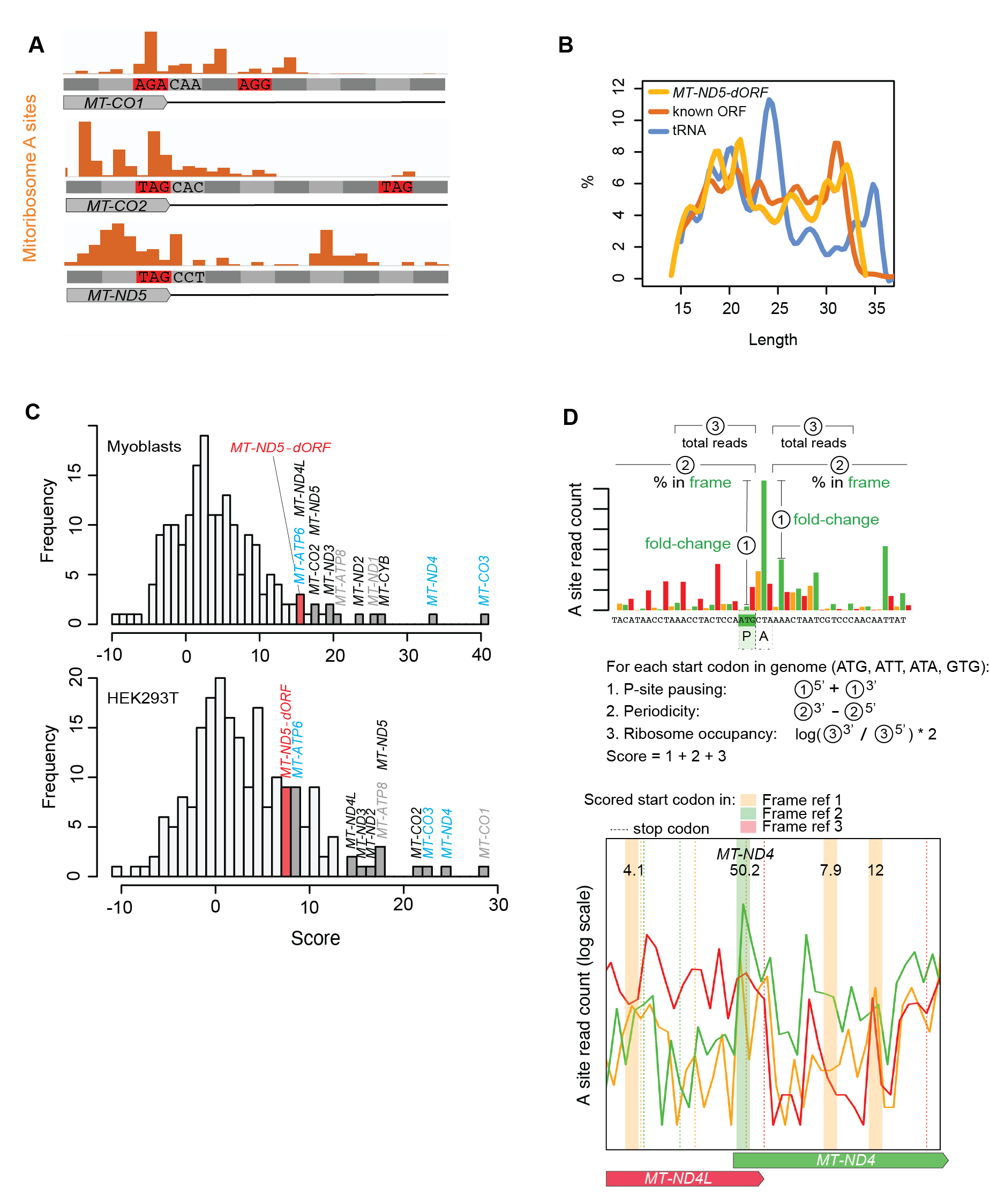


**Figure S2**. **Translation of a novel mitochondrial open reading frame**. (**A**) RPF density on stop codons and into the 3’ UTRs of *MT-CO1*, *MT-CO2*, and *MT-ND5*. Stop codons are marked in red and the trinucleotide sequence following each stop codon is labeled. (**B**) Read length distribution for all reads across a long version of MT-ND5-dORF (19 codons, extending to the next downstream stop codon), a randomly chosen region of the same length in *MT-ND5*, and the tRNA *MT-TL2*. (**C**) Ad hoc scoring strategy to identify likelihood of translation initiation at each start codon. Top panel: Details of scoring computation. Windows used in {2} are 16 codons upstream and 20 codons downstream. Window used in {3} is five codons. See methods for more details. Bottom panel: example output including region of raw data shown above. Wide vertical lines show putative start codons with their scores, with the color indicating their frame. Dotted vertical lines show putative stop codons, with the color indicating their frame. The number at top are the ad hoc scores. (**D**) Ad hoc score distributions, as in Figure 2C, for additional cell lines. Gene names in black: known genes with no leader; gray: known genes with 1 to 3 nt leader; cyan: known genes with long 5’ UTR.


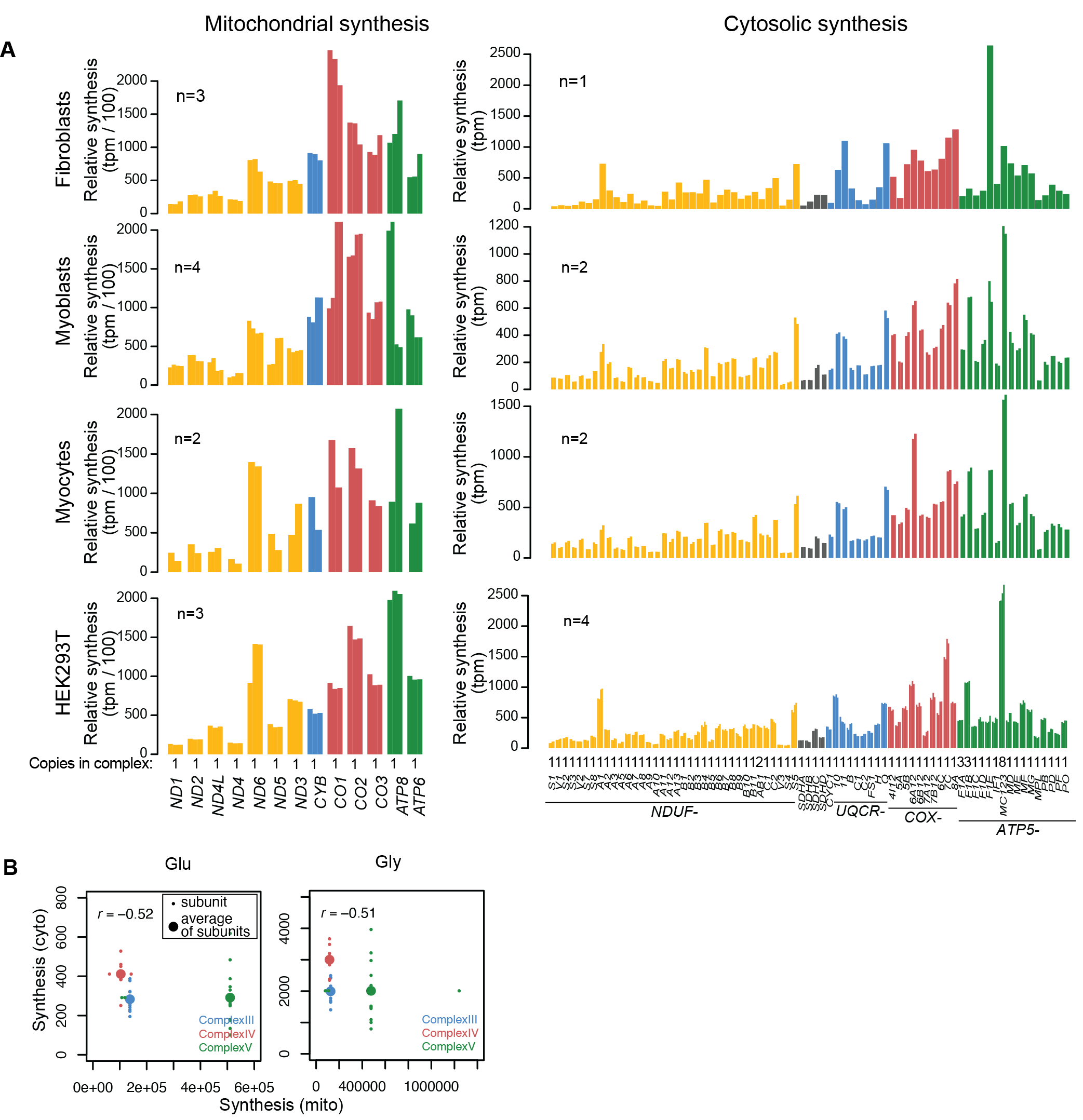


**Figure S3**. **Correlated synthesis of mtDNA- and nDNA-encoded OXPHOS subunits.** (**A**) All data included in Figure 3C, D. Each replicate is shown as an individual bar, values given in **Additional File 3: Table S2**. Cytosolic synthesis values for fibroblasts were calculated from cytosolic ribosome profiling data from (Tirosh et al, 2015). Relative synthesis is measured in tpm/100 for mitochondrial synthesis and tpm for cytosolic synthesis (see Methods for details and note that mitochondrial and cytosolic synthesis absolute values cannot be compared across compartments). (**B**) Synthesis of *S. cerevisiae* mtDNA-encoded OXPHOS subunits (RPKM) compared to synthesis of nDNA-encoded subunits (RPKM) for each complex. Values taken from [[12]](https://paperpile.com/c/WVkvpP/Iig8h). Small dots show individual subunits, large dots show average synthesis of subunits within each corresponding complex, following the color code displayed.


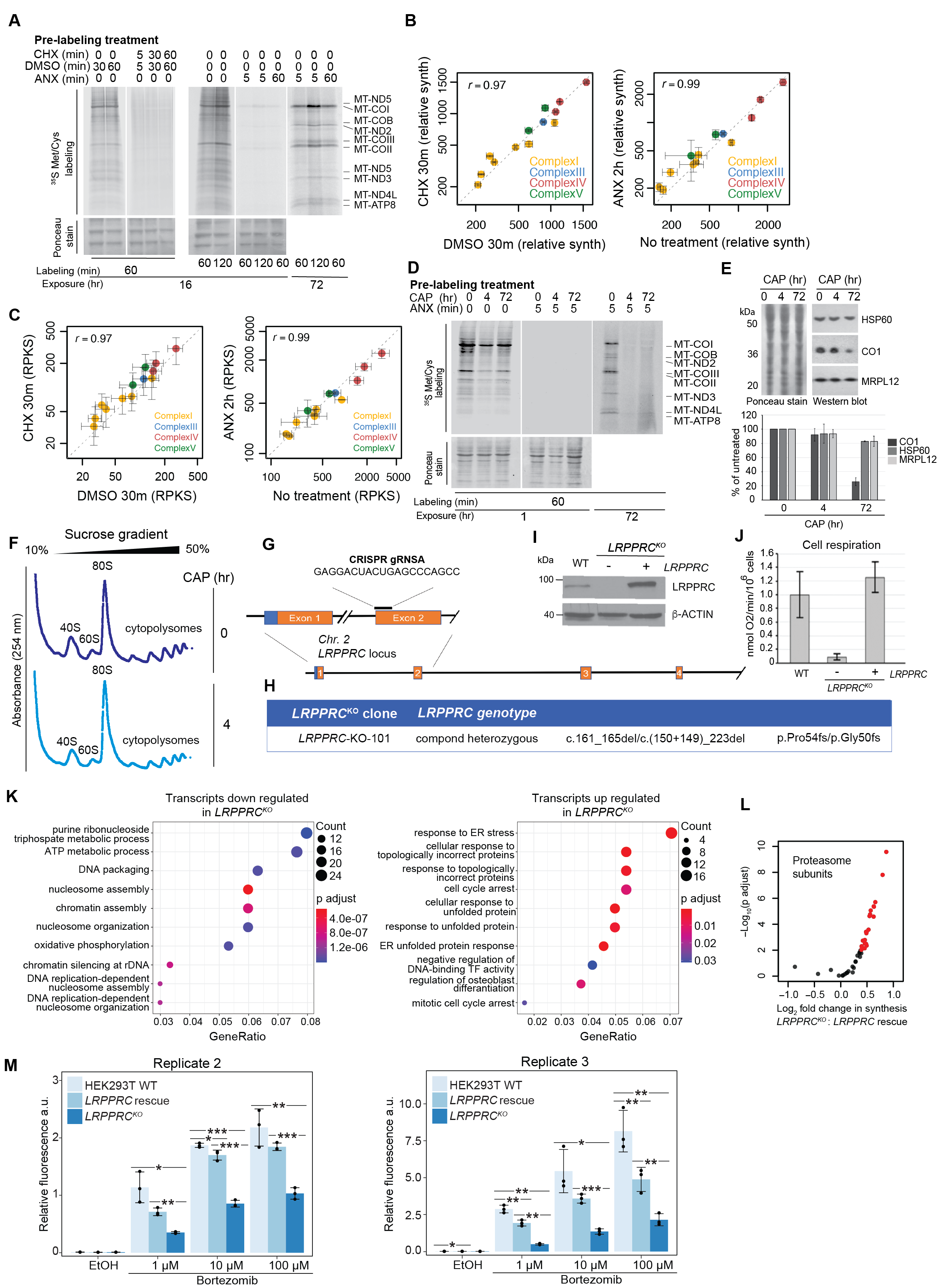


**Figure S4.** **Translation perturbations.** (**A**) *In vivo* labeling of translation products. Human fibroblasts newly synthesized products were metabolically labeled with ^35^S methionine/cysteine (top panels), after treatment with either cycloheximide (CHX), anisomycin (ANX) or DMSO control, for the indicated times. Signals were exposed to a screen for 16 or 72 hrs, as explained in Methods. Sample loading was followed by Ponceau stain (bottom panels). (**B**) Comparison of relative synthesis values (tpm/100) for mtDNA-encoded OXPHOS subunits with and without 30 minutes of cycloheximide (CHX) (100 µg/mL) or 2 hours of anisomycin (ANX) treatment (100 µg/mL) to inhibit cytosolic translation. (**C**) Comparison of RPKS (mouse spike-in normalized reads per kb) values for mtDNA-encoded OXPHOS subunits with and without 30 minutes of cycloheximide (CHX) (100 µg/mL) or 2 hours of anisomycin (ANX) treatment (100 µg/mL) to inhibit cytosolic translation. (**D**) *In vivo* labeling of translation products. Human fibroblasts newly synthesized products were metabolically labeled with ^35^S-methionine/cysteine (top panels), after treatment with chloramphenicol (CAP) for the indicated times, while in the presence of cytosolic translation inhibitor anisomycin (ANX). Signals were exposed to a screen for 1 or 72 hrs, as explained in Methods. Sample loading was followed by Ponceau stain (bottom panels). (**E**) Western blot analysis of HEK293T cells treated with chloramphenicol (CAP) for the indicated times (top panel). Protein marker molecular weights are indicated in kDa. Quantification shows the signal percentage of untreated cells normalized by Ponceau stain (lower panel). (**F**) Sedimentation of cytosolic polysomes in 10-50% linear sucrose gradients. HEK293T cells were treated with mitochondrial ribosome inhibitor chloramphenicol (CAP) for the indicated times in hours (hr). Cells were subsequently lysed and loaded onto gradients as explained in the methods section for cytoribosome profiling. Cytoribosomes were detected by their UV absorbance at 254 nm. (**G**) Schematic showing the location of the target sites in the *LRPPRC* locus for the CRISPR guide RNA used to knock out the gene. (**H**) Table detailing the genotype of the generated HEK293T cell line carrying edited *LRPPRC* alleles. (**I**) Immunoblot analysis of LRPPRC steady-state levels in WT and *LRPPRC*^KO^ cells and *LRPPRC*^KO^ cells reconstituted with recombinant *LRPPRC*. An antibody against b-actin was used as the loading control. Protein marker molecular weights are indicated in kDa. (**J**) Endogenous cell respiration in WT, *LRPPRC*^KO^, and WT rescue cell lines measured polarographically. (**K**) GO-term enrichment analysis of significantly (adjusted p-value < 0.05) differentially expressed genes that are at least two-fold decreased or increased in the *LRPPRC*^KO^ cells compared to the WT rescue cell line*.* Count = number of genes sharing a GO-term, p adjust = adjusted p-value, TF = transcription factor. (**L**) Volcano plot showing differential synthesis of proteasome subunits (HGNC gene group Proteasome (PSM), <https://www.genenames.org/data/genegroup/#!/group/690>). Values were calculated using DESeq2, comparing cytoribosome profiling data from *LRPPRC^KO^* to *LRPPRC* rescue cell lines. Significantly differentially expressed genes (adjusted p-value < 0.01) are shown in red. (**M**) The graphs represent two biological replicates of the cell toxicity assay. Replicate 1 is presented in Figure 4G. Cells were treated for 72 hours with increasing amounts of bortezomib or the solvent ethanol. Cell toxicity was measured with a fluorescent dye and normalized to the number of living cells measured by luminescence. Each measurement contained 3 technical replicates and the range bars show the standard deviation. The Welch two sample one sided (less) t test was used to test for significant changes. * signifies p-value < 0.05, ** signifies p-value < 0.01 and *** signifies p-value < 0.001.
