# Supplementary material for: Balanced mitochondrial and cytosolic translatomes underlie the biogenesis of human respiratory complexes
Source: Genome Biol. 2022 Aug 9;23:170. doi: 10.1186/s13059-022-02732-9 (PMC9361522; doi:10.1186/s13059-022-02732-9)
Supplement: Supplementary file 6 — Additional file 6. Review History. [file 13059_2022_2732_MOESM6_ESM.docx]

Review History

**First round of review**

**Reviewer 1**

**Were you able to assess all statistics in the manuscript, including the appropriateness of statistical tests used?**

Yes

**Comments to author:**

Soto, Couvillion, and colleagues report here on translational profiling of human mitochondria. Following the conceptual approach used previously by the same group to study yeast mitochondrial translation, a mitoribosomal protein is tagged to allow affinity purification of the mitoribosome. Extensive validation data is provided to support the procedure for mitochondrial ribosome footprint generation and sequencing. These data are argued to reveal translation of a short reading frame in the 3' UTR of a mitochondrial transcript, MT-ND5. Quantitative translation measurements are then used to study the synthesis of oxidative phosphorylation (OXPHOS) complexes that are assembled from cytosolically and mitochondrially translated subunits. OXPHOS complexes show balanced synthesis—with closer balance at the level of translation than mRNA abundance. This balance does not seem to depend on any rapid regulation between cytosolic and mitochondrial translation, however, in contrast to the situation described previously by the same researchers for yeast. Disruptions of cytosolic translation do not affect mitochondrial protein synthesis. Likewise, derangement of mitochondrial translation by the deletion of the nuclear-encoded regulatory protein LRPPRC does not affect synthesis of cytosolic OXPHOS proteins. Dysregulation of OXPHOS protein synthesis does appear to activate the unfolded protein response, however; it also renders cells less sensitive to the proteasome inhibitor bortezomib.

The mitochondrial ribosome profiling approach reported here is clearly a technical advance. The resulting data provide a clearer and more quantitative picture of mitochondrial translation. It is notable that coordination of mitochondrial and cytosolic translation in human cells must rely on different mechanisms than in yeast. Although the regulatory mechanisms remain to be determined, the importance of this regulation is highlighted by the stress responses caused by LRPPRC deletion. The new view of mitochondrial translation provided here will interest the readership of Genome Biology and I would support its publication, although I do have specific concerns.

1. Ribosome density is reported in the MT-ND5 3' UTR (Figure 2). While a metric is developed to assess potential sites of translation initiation, there is not so much evidence for elongating ribosomes across this ORF. Most samples don't show ribosome occupancy past this single potential start site, though (Figure 2A). When ribosomes are seen on the ORF, they extend past the stop codon, leading to the proposal that read-through explains the pattern of ribosome occupancy. Given the fundamental differences between mitochondrial and cytosolic translation, the observation of a "context that promotes read-through in the cytosol" is irrelevant, and in any case, the absolute amount of read-through is very low in the absence of G418 treatment. The data presented don't robustly support translation of the specific proposed ORF or exclude other alternatives, like protection by an RNA-binding protein.

2. The effects of LRPPRC deletion are seen to trigger a "response to ER stress". However, there is substantial overlap between the responses to ER stress and responses to mitochondrial stress, including those mediated by the OMA1-DELE1-HRI pathway. Is it possible to determine which branches of the UPR are activated by LRPPRC deletion and to test whether there is dependency on PERK or alternatively HRI?

3. Bortezomib resistance is not the expected result of impaired proteostasis. The more straightforward model would produce a synergistic enhancement of bortezomib sensitivity. The hormesis model proposed here, where activation of ER stress responses would be protective against bortezomib, is one possible explanation.

Some technical explanations also arise, however. Bortezomib effects are assessed by an assay for dead cells carried out at one timepoint. Bortezomib induces apoptosis through the mitochondrial pathway (doi:10.1038/sj.onc.1210744), and the dysfunction produced by LRPPRC knock-out could interfere with apoptosis and distort this measurement. Alternately, if LRPPRC knock-out cells grow more slowly, they could be functionally more resistant to Bortezomib.

**Reviewer 2**

**Were you able to assess all statistics in the manuscript, including the appropriateness of statistical tests used?**

Yes

**Comments to author:**

Soto et al. perform a systematic analysis of mitochondrial translatomes in human cells by ribosome footprint to investigate mechanisms regulating the biogenesis of mitochondrial complexes with a dual genetic origin. Based on a previous study in budding yeast, the authors hypothesised that OXPHOS biogenesis would occur through synchronous translation programs and rapid feedback between the cytosolic and mitochondrial translation machineries. Instead, they highlight substantial differences in mito-nuclear balance between fungal and animal cells and vulnerabilities of the latter to proteotoxicity to precisely balance cytosolic and mitochondrial translatomes. Overall, the experimental techniques and methods used to investigate coupled mitochondrial and cytosolic translatomes are innovative and appropriate and include well-thought control conditions.

Their conclusions are adequately supported by the data and considering the broad interest of this study also to other fields focusing on inter-organelles signalling and more generally on biogenesis of multiprotein complexes I find it suitable for publication in Genome Biology.

**Authors Response**

**Point-by-point responses to the reviewers’ comments:**

*We thank the reviewers for their comments and are pleased that they support the publication of our manuscript. We performed additional computational and experimental analyses in response to the reviewers’ concerns that are described below. We have edited the manuscript to incorporate these new results and to clarify our findings.*

Reviewer #1: Soto, Couvillion, and colleagues report here on translational profiling of human mitochondria. Following the conceptual approach used previously by the same group to study yeast mitochondrial translation, a mitoribosomal protein is tagged to allow affinity purification of the mitoribosome. Extensive validation data is provided to support the procedure for mitochondrial ribosome footprint generation and sequencing. These data are argued to reveal translation of a short reading frame in the 3' UTR of a mitochondrial transcript, MT-ND5. Quantitative translation measurements are then used to study the synthesis of oxidative phosphorylation (OXPHOS) complexes that are assembled from cytosolically and mitochondrially translated subunits. OXPHOS complexes show balanced synthesis—with closer balance at the level of translation than mRNA abundance. This balance does not seem to depend on any rapid regulation between cytosolic and mitochondrial translation, however, in

contrast to the situation described previously by the same researchers for yeast. Disruptions of cytosolic translation do not affect mitochondrial protein synthesis. Likewise, derangement of mitochondrial translation by the deletion of the nuclear-encoded regulatory protein LRPPRC does not affect synthesis of cytosolic OXPHOS proteins. Dysregulation of OXPHOS protein synthesis does appear to activate the unfolded protein response, however; it also renders cells less sensitive to the proteasome inhibitor bortezomib.

The mitochondrial ribosome profiling approach reported here is clearly a technical advance. The resulting data provide a clearer and more quantitative picture of mitochondrial translation. It is notable that coordination of mitochondrial and cytosolic translation in human cells must rely on different mechanisms than in yeast. Although the regulatory mechanisms remain to be determined, the importance of this regulation is highlighted by the stress responses caused by LRPPRC deletion. The new view of mitochondrial translation provided here will interest the readership of Genome Biology and I would support its publication, although I do have specific concerns.

*Response: We thank the reviewer for the summary and the positive comments regarding our mitochondrial translation work.*

1. Ribosome density is reported in the MT-ND5 3' UTR (Figure 2). While a metric is developed to assess potential sites of translation initiation, there is not so much evidence for elongating ribosomes across this ORF. Most samples don't show ribosome occupancy past this single potential start site, though (Figure 2A). When ribosomes are seen on the ORF, they extend past the stop codon, leading to the proposal that read-through explains the pattern of ribosome occupancy. Given the fundamental differences between mitochondrial and cytosolic translation, the observation of a "context that promotes read-through in the cytosol" is irrelevant, and in any case, the absolute amount of read-through is very low in the absence of G418 treatment. The data presented don't robustly support translation of the specific proposed ORF or exclude other alternatives, like protection by an RNA-binding protein.

*Response: To address these comments, we have analyzed the lengths of reads that map to the MT-ND5-dORF to test whether they are likely derived from ribosome-protected fragments or an alternative source. This analysis is now shown in Figure S2B with the following text added to the manuscript:*

*To determine whether the MT-ND5 downstream ORF (MT-ND5-dORF) is likely to be translated, we first investigated the distribution of the lengths of reads that map to the ORF. We compared the read length distribution to a region of the same size in a known ORF and across a tRNA (Figure S2B). The lengths of reads mapping to MT-ND5-dORF are not significantly different from those mapping to known coding regions (Kolmorgorov-Smirnov test, p=0.136) but are significantly different from those mapping across tRNA (Kolmorgorov-Smirnov test, p=0.000279), suggesting that they are derived from ribosome protection and not protection from another RNA binding protein or structured/double-stranded RNA.*

*In addition, we have added rationale for why translational read-through might be occurring at MT-ND5-dORF with the following text: “we observe abundant read-through of MT-CO1, MT-CO2, and MT-ND5, three of the four messages that have true 3’ UTRs (Figure S2A). At each of these, as well as at the short ORF, the stop codon is followed by a C, a context that promotes read-through in the cytosol.” Supporting this statement, we have added a figure panel showing read-through at all three genes (Figure S2A). Finally, we have edited the text throughout to clarify and soften our conclusions.*

2. The effects of LRPPRC deletion are seen to trigger a "response to ER stress". However, there is substantial overlap between the responses to ER stress and responses to mitochondrial stress, including those mediated by the OMA1-DELE1-HRI pathway. Is it possible to determine which branches of the UPR are activated by LRPPRC deletion and to test whether there is dependency on PERK or alternatively HRI?

*Response: We agree. Although the analysis displayed in Figure 4F uses the official GO-term “ER stress”, we avoided describing the stress response as ER or mitochondrial in the text due to the points that the reviewer makes here. Because the knockout cells are in a late stage of any response, it is not possible to recapitulate the activation events that occurred when the gene was first deleted. Nevertheless, we appreciate that that more insight into the response would enrich our results, so we performed western blot analysis of key factors for the branches of the UPR to gain more insight into the response we observe. We find evidence for a complex adaptive response in these cells (increased phosphorylated IRE1, CHOP and ATF3, decreased ATF4 and ATF6) (Figure 4H). Our data cannot distinguish between a PERK or HRI dependent pathway without performing a time-series analysis from the onset of LRPPRC deletion. However, we observe an increase in mtHSP70, indicating that the cells have responded to mitochondrial stress. We have added these western blot results to Figure 4 and described them in the text.*

3. Bortezomib resistance is not the expected result of impaired proteostasis. The more straightforward model would produce a synergistic enhancement of bortezomib sensitivity. The hormesis model proposed here, where activation of ER stress responses would be protective against bortezomib, is one possible explanation.

Some technical explanations also arise, however. Bortezomib effects are assessed by an assay for dead cells carried out at one timepoint. Bortezomib induces apoptosis through the mitochondrial pathway (doi:10.1038/sj.onc.1210744), and the dysfunction produced by LRPPRC knock-out could interfere with apoptosis and distort this measurement. Alternately, if LRPPRC knock-out cells grow more slowly, they could be functionally more resistant to Bortezomib.

*Response: We reanalyzed our data to identify what might account for the Bortezomib resistance in LRPPRC^KO^ cells. Interestingly, we found that these cells show higher synthesis of proteasomal subunits compared to WT cells (Figure S4L), which could explain their improved tolerance of a proteasome inhibitor. Nevertheless, in light of the reviewer’s comments, we have softened our conclusions on proteostasis and have focused on the upregulation of stress response genes. We now state:*

*Additionally, LRPPRC^KO^ cells were less sensitive to bortezomib, a proteasome inhibitor, consistent with the higher synthesis of proteasomal subunits in these cells (Figure S4L). We have interpreted these results as indicating that the induction of the protein quality control genes may offer protection from further proteotoxicity (Figure 4G, Figure S4M). In sum, loss of LRPPRC disrupts mitonuclear translational balance and promotes the upregulation of proteostasis pathways.*

Reviewer #2: Soto et al. perform a systematic analysis of mitochondrial translatomes in human cells by ribosome footprint to investigate mechanisms regulating the biogenesis of mitochondrial complexes with a dual genetic origin. Based on a previous study in budding yeast, the authors hypothesised that OXPHOS biogenesis would occur through synchronous translation programs and rapid feedback between the cytosolic and mitochondrial translation machineries. Instead, they highlight substantial differences in mito-nuclear balance between fungal and animal cells and vulnerabilities of the latter to proteotoxicity to precisely balance cytosolic and mitochondrial translatomes. Overall, the experimental techniques and methods used to investigate coupled mitochondrial and cytosolic translatomes are innovative and appropriate and include well-thought control conditions.

Their conclusions are adequately supported by the data and considering the broad interest of this study also to other fields focusing on inter-organelles signalling and more generally on biogenesis of multiprotein complexes I find it suitable for publication in Genome Biology.

*Response: We thank the reviewer for the positive comments.*

**Second round of review**

**Reviewer 1**

Revisions have fully addressed concerns with the original submission. New data on the complex adaptive response to LRPPRC add to the manuscript, as do the analyses of translation and read-through on MT-ND5 and elsewhere.
